# The tumour suppressor DLC2 ensures mitotic fidelity by coordinating spindle positioning and cell–cell adhesion

**DOI:** 10.1038/ncomms6826

**Published:** 2014-12-18

**Authors:** Elisa Vitiello, Jorge G. Ferreira, Helder Maiato, Maria S. Balda, Karl Matter

**Affiliations:** 1 Department of Cell Biology, UCL Institute of Ophthalmology, University College London, London EC1V 9EL, UK; 2 Chromosome Instability & Dynamics Laboratory, Instituto de Biologia Molecular e Celular, Universidade do Porto, Rua do Campo Alegre 823, 4150-180 Porto, Portugal; 3 Cell Division Unit, Department of Experimental Biology, Faculty of Medicine, University of Porto, 4200-319 Porto, Portugal

## Abstract

Dividing epithelial cells need to coordinate spindle positioning with shape changes to maintain cell–cell adhesion. Microtubule interactions with the cell cortex regulate mitotic spindle positioning within the plane of division. How the spindle crosstalks with the actin cytoskeleton to ensure faithful mitosis and spindle positioning is unclear. Here we demonstrate that the tumour suppressor DLC2, a negative regulator of Cdc42, and the interacting kinesin Kif1B coordinate cell junction maintenance and planar spindle positioning by regulating microtubule growth and crosstalk with the actin cytoskeleton. Loss of DLC2 induces the mislocalization of Kif1B, increased Cdc42 activity and cortical recruitment of the Cdc42 effector mDia3, a microtubule stabilizer and promoter of actin dynamics. Accordingly, DLC2 or Kif1B depletion promotes microtubule stabilization, defective spindle positioning, chromosome misalignment and aneuploidy. The tumour suppressor DLC2 and Kif1B are thus central components of a signalling network that guides spindle positioning, cell–cell adhesion and mitotic fidelity.

## Abstract (web summary)

Epithelial cells must position their mitotic spindle correctly to maintain cell–cell adhesion. Here Vitiello *et al*. show that the tumour suppressor DLC2 and the mitotic kinesin Kif1b coordinate microtubule–actin interactions upstream of mDia3, guiding spindle positioning and mitotic fidelity.

## Graphical abstract

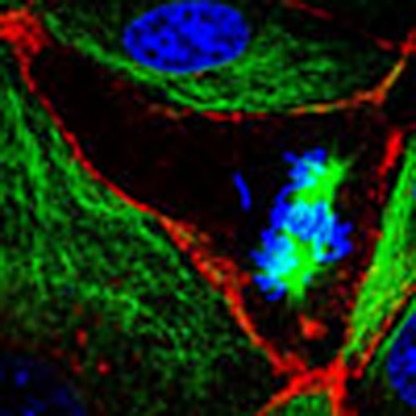

Epithelial development, maintenance and repair requires that cells can divide and adapt to complex cell shape changes without dissociating their contacts with neighbouring cells and, hence, that they can sense how to position their mitotic spindle^1^. Spindle positioning is determined by astral microtubules that originate at the spindle poles and extend towards the cell cortex where they are thought to interact with actin structures that transmit extracellular cues^2^. However, it is poorly understood how astral microtubules are regulated to ensure proper spindle positioning and whether such mechanisms also affect cell–cell adhesion to maintain the tissue integrity during mitotic cell shape changes.

In mammals, epithelial cell–cell adhesion is mediated by three types of junctions: tight junctions, adherens junctions and desmosomes, which form the epithelial junctional complex^3^,^4^,^5^,^6^,^7^. Junction maintenance and coordinated remodelling are fundamental to preserve an intact tissue during cell shape changes and are mainly driven by cortical actin dynamics^8^. During epithelial cell division, junctions represent a reference point to guide the positioning of the mitotic spindle and division^9^, and to anchor the mitotic spindle^10^. However, such mechanisms require astral microtubules to grow to the appropriate size to position the mitotic spindle correctly. How this is regulated is not clear. Similarly, whether mechanisms that regulate astral microtubule growth also affect cell–cell junctions is unknown.

The small GTPase Cdc42 plays a major role in epithelial tissue formation and homeostasis. Cdc42 cycles between an active state (GTP bound) and inactive state (GDP bound), and its regulation is controlled by factors that either mediate guanine nucleotide exchange or that stimulate GTP hydrolysis (GAPs). Tight regulation of Cdc42 is crucial for junction formation and maintenance, as well as for mitotic spindle positioning and chromosome attachment^11^,^12^,^13^,^14^,^15^,^16^,^17^,^18^. Temporal and spatial control of Cdc42 during junction formation and maintenance is thought to require GEFs to drive activation and GAPs to ensure appropriate termination of the signal and spatial control to ensure junctional stabilization^11^,^19^,^20^,^21^,^22^. Similarly, regulators have been identified that guide spindle orientation^23^,^24^,^25^. However, how Cdc42 is controlled to guide spindle positioning (that is, the location and orientation within the plane of the tissue), and the molecular and cellular processes and principles that it controls during this process are not well understood.

Here we show that the tumour suppressor DLC2, a GAP (GTPase-activating protein) for Cdc42, regulates microtubule growth and cortical actin polarization, and, thereby, coordinates spindle positioning and junctional integrity. DLC2 cooperates with the new mitotic kinesin Kif1B and functions upstream of mDia3, a Cdc42 effector that regulates microtubule and actin organization. The DLC2 regulatory network is also required for mitotic fidelity, providing a molecular explanation for its tumour suppressor activity.

## Results

### DLC2 regulates chromosome alignment

**Figure 1:**
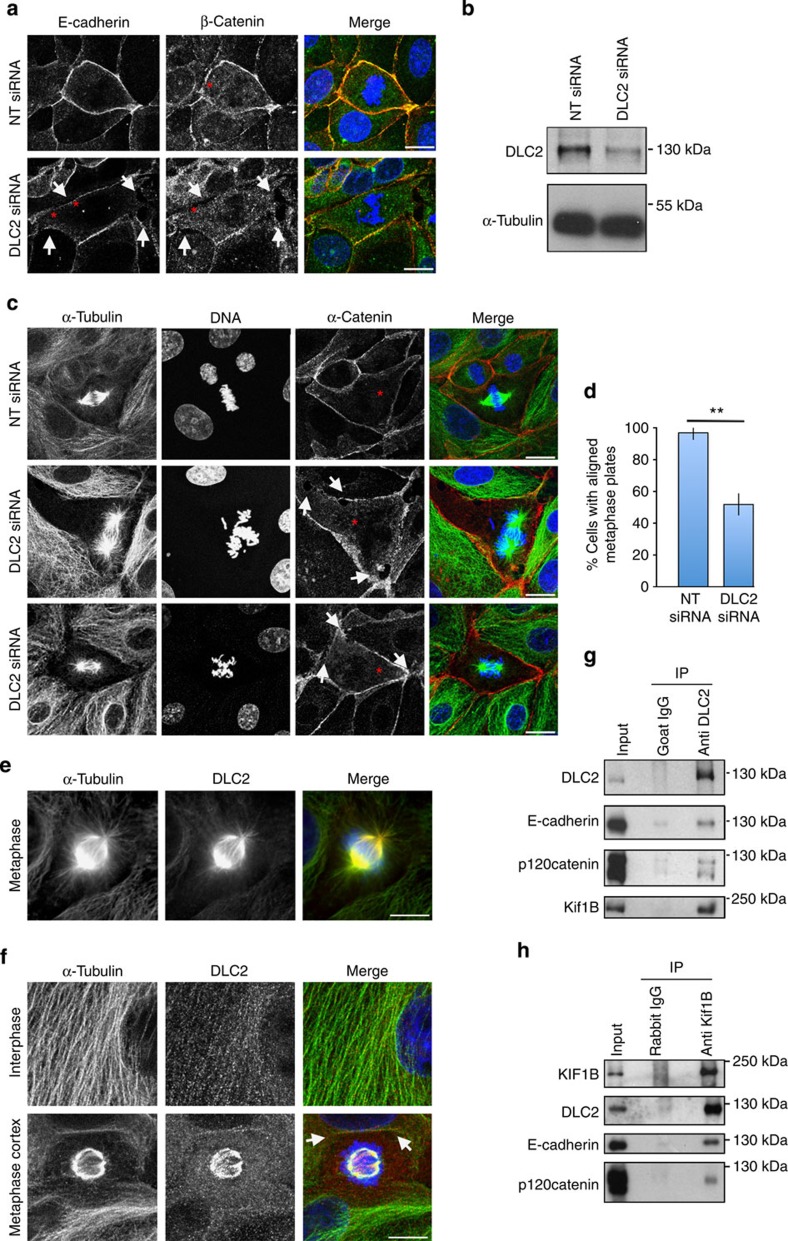
DLC2 is required for junction maintenance and mitotic spindle stability. (**a**) Control and DLC2-depleted HCE cells stained for the adherens junction proteins E-cadherin (red) and β-catenin (green) and DNA (blue). (**b**) Immunoblot for DLC2 of control and DLC2 siRNA-transfected HCE cells; α-tubulin was used as a loading control. (**c**) Control and DLC2-depleted HCE cells stained for α-tubulin (green), α-catenin (red) and DNA (blue). (**d**) Quantification of cells with bipolar spindles with aligned and misaligned metaphase plates after transfection of control and DLC2 siRNAs (shown are means±1 s.d.; **P<0.01 (*t*-test); *n*=3 experiments; at least 60 mitotic cells were counted per condition in each of these three experiments). See Supplementary Fig. 1 for the deconvolution of the DLC2 siRNA pool (**e**,**f**) HCE cells stained for α-tubulin (green), DLC2 (red) and DNA (blue). (**g**) HCE cell extracts were immunoprecipitated with goat anti-DLC2 IgG or control goat IgGs conjugated directly to Protein G-Sepharose and precipitates were immunoblotted as indicated. (**h**) Cell extracts were immunoprecipitated with rabbit anti-Kif1B antibodies or control rabbit IgG conjugated to beads and analysed by immuoblotting with the indicated antibodies. (**a**,**c**) Mitotic cells are labelled with an asterisk and locally disrupted cell–cell contacts with arrows. In **f**, the arrows point to the labelled cell cortex. Scale bars, 10 μm.

Depletion of DLC2 (Deleted in Liver Cancer 2; STARD13) in interphase cells has only a mild effect on junction formation in differentiating human epithelial cells^21^. However, mitotic cells exhibited striking defects in integrity of tight and adherens junctions with gaps appearing between neighbouring human corneal epithelial (HCE) cells, one of the cell lines used for the short interfering RNA (siRNA) screen described by Elbediwy *et al*.^21^ (Fig. 1a,b and Supplementary Fig. 1a). HCE cells form regular monolayers with fully assembled cell–cell junctions and can be efficiently transfected with siRNAs. Depletion also affected the arrangement of chromosomes within the mitotic spindle. A fraction of mitotic cells formed bipolar spindles reminiscent of metaphase cells, as the chromosomes were more clustered than in prometaphase, but they remained poorly aligned and the spindle axis was often not perpendicular to the metaphase plate (Fig. 1c,d). Control cells transitioned efficiently from prometaphase to metaphase (more than 85% of all cells in prometaphase and metaphase had well aligned metaphase plates). Hence, DLC2 depletion affected the transition from prometaphase to metaphase or maintenance of the metaphase plate.

To test the specificity of the siRNA depletion, we tested individual siRNAs for their phenotype. Supplementary Fig. 1c–f shows that the three individual siRNAs affected metaphase plate formation in a similar way as the siRNA pool and also led to reduced junctional p120 catenin staining in mitotic cells. We could not test the specificity by complementation as transfection of DLC2 inhibited G1 exit as previously published^26^. Hence, DLC2 seems to regulate spindle assembly, possibly involving interplay between the spindle and the cell cortex. DLC2 was first reported as a tumour suppressor in liver cancer and is repressed in various other cancers^27^,^28^,^29^. Hence, the loss of junctional integrity and defects in the mitotic apparatus may contribute to the disease.

By immunofluorescence, DLC2 localizes along microtubules (Fig. 1e,f). Anti-DLC2 antibodies labelled spindle fibres and, weakly, astral microtubules, and this staining was strongly reduced in DLC2 depleted cells (Fig. 1e,f and Supplementary Fig. 1g,h). When DLC2 was depleted, membrane association of APC, a mediator of microtubule anchorage at the cell cortex^30^, was reduced (Supplementary Fig. 1b). Therefore, DLC2 knockdown might affect microtubule interactions with cell junctions.

### DLC2 associates with Kif1B

We next asked whether DLC2 interacts with proteins associated with microtubules and cell junctions. A report based on a two-hybrid screen has previously suggested that DLC2 can interact directly with α-tubulin and Kif1B (ref. 31). Indeed, immuoprecipitation of DLC2 from HCE cell extracts resulted in the co-immunoprecipitation of Kif1B (Fig. 1g). Moreover, the two adherens junction proteins p120 catenin and E-cadherin were also recovered in the precipitates. Similarly, immunoprecipitates of Kif1B contained DLC2, p120 catenin and E-cadherin (Fig. 1h). Hence, DLC2 forms a complex with the kinesin Kif1B, and the two proteins can interact with adherens junction proteins.

**Figure 2:**
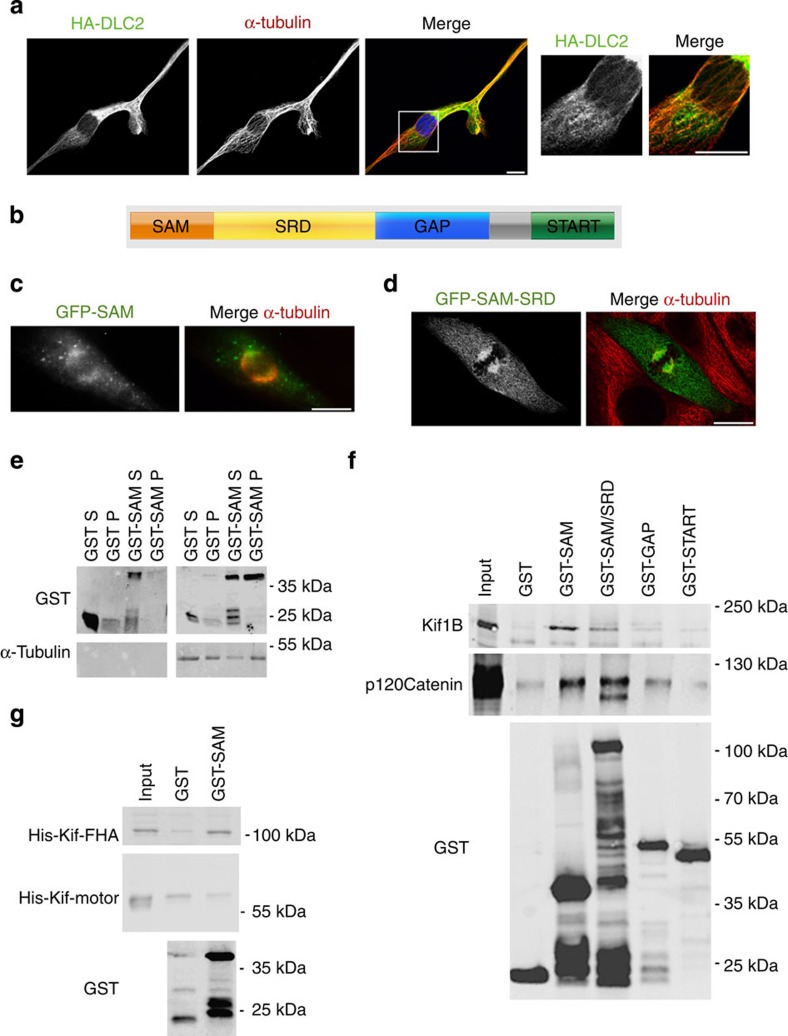
DLC2 interacts with microtubules. (**a**) HCE cells were transfected with haemagglutinin (HA)-tagged DLC2 and stained for the transfected protein and microtubules. The box marks the area magnified in the panels on the right. (**b**) Domain structure of DLC2. Indicated are the sterile alpha motif (SAM), the serine-rich domain (SRD), the GAP and the steroidogenic acute regulatory protein-related lipid-transfer domain (START). (**c**,**d**) HCE cells were transfected with the indicated DLC2 domain constructs fused to GFP and localized in cells labelled for α-tubulin. Shown are mitotic cells; see Supplementary Fig. 2 for localization in interphase cells. (**e**) Binding of the SAM domain to microtubules was tested using a tubulin co-sedimentation assay. The SAM domain was purified as a GST-fusion protein and GST alone was used as a negative control. The GST-fusion proteins (20 μg ml^−1^) were incubated with taxol-stabilized microtubules (0.45 mg ml^−1^) before centrifugation. Centrifugation in the absence of tubulin was used to test solubility of the fusion proteins. Shown are the immunoblots using antibodies against GST to detect the recombinant proteins and α-tubulin (**f**) Binding of DLC2 domains to Kif1B was tested in pull-down assays using HCE cell extracts and GST-fusion proteins containing the indicated domains bound to beads. The pull downs were then immunoblotted for Kif1B, p120 catenin and GST. (**g**) Interaction between the DLC2 SAM domain fused to GST and recombinant His-tagged fusion proteins containing the Kif1B motor or FHA domain was tested in pull-down assays using purified GST proteins bound to beads. The precipitates were then analysed by immunoblotting using anti-GST and anti-His antibodies. Scale bars, 10 μm.

The partial association of endogenous DLC2 with microtubules and its association with mitotic spindles (Fig. 1e,f) suggested that it might bind microtubules directly. The above-mentioned two-hybrid screen also supports this possibility^31^. To test this, we first transfected full-length and truncated DLC2 into HCE cells. Figure 2a shows that the full-length construct strongly affected cell morphology, presumably because of its GAP activity, but also partially co-localized with microtubules. DLC2 contains four main structural domains (Fig. 2b). Green fluorescent protein (GFP)-tagged constructs containing the amino-terminal SAM domain associated with cell borders in interphase cells, but were recruited to spindles during mitosis (Fig. 2c,d and Supplementary Fig. 2a). A SAM domain construct was also found to co-sediment with microtubules, further supporting the conclusion that the SAM domain can link DLC2 to microtubules (Fig. 2e).

As the SAM domain constructs could target cell borders, we asked whether this domain also links DLC2 to adherens junction proteins. Figure 2f shows that pull downs with GST-fusion proteins containing the SAM domain contained Kif1B as well as p120 catenin (Fig. 2f). A His-tagged recombinant protein containing the forkhead-associated (FHA) domain of Kif1B was also found to interact with DLC2, whereas one containing the kinesin motor domain did not (Fig. 2g). These data are in agreement with the two-hybrid results, indicating that DLC2 and Kif1B can interact directly. The expression of SAM domain constructs and the Kif1B FHA domain led to the formation of peripheral microtubule bundles and gaps between cells, suggesting that DLC2 and Kif1B might regulate microtubules (Supplementary Fig. 2c).

### DLC2 regulates Cdc42 during mitosis

**Figure 3:**
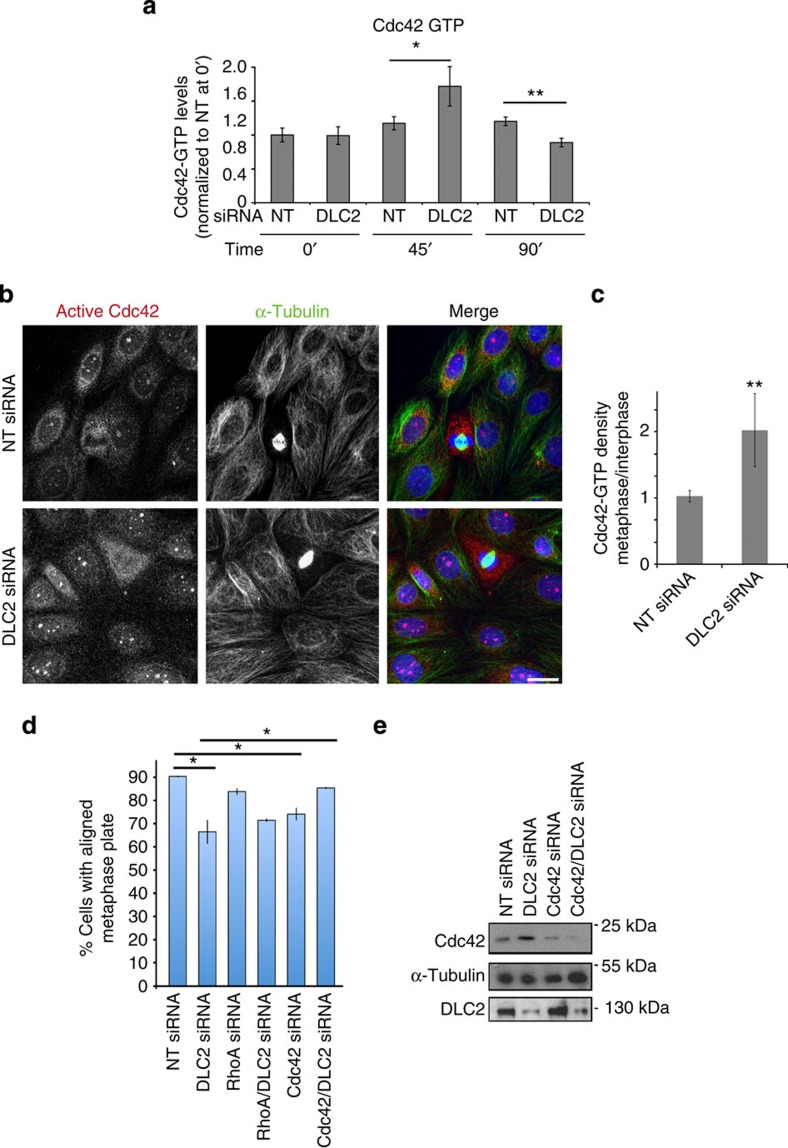
DLC2 regulates Cdc42 in metaphase. (**a**) Cdc42 activity assays of control (NT, non-targeting) and DLC2 siRNA-transfected HCE cells that had been synchronized with Nocodazole. Time points analysed after Nocodazole washout were 0, 45 and 90 min (shown are means±1 s.d.; *n*=3 experiments; 45-min washout corresponds to metaphase cells; values were normalized to control cells at time 0). (**b**) HCE cells transfected with control and DLC2 siRNAs were stained with antibodies against active Cdc42 and α-tublin, as well as DNA (blue). (**c**) Quantification of fluorescence intensity was performed by calculating the ratio of mean intensities measured in metaphase and interphase cells (shown are means±1 s.d.; **P<0.01; *n*=3 experiments). (**d**) Count of aligned metaphase plates. HCE cells were transfected with non-targeting, DLC2, Cdc42, RhoA, Cdc42 and DLC2, RhoA and DLC2 siRNAs. The cells were then fixed and stained for α-tubulin and DNA, and cells with aligned and misaligned metaphase plates were counted (shown are means±1 s.d.; *n*=3 experiments). (**e**) Total extracts of HCE cells transfected with siRNAs as indicated were analysed by immunoblotting for Cdc42, DLC2 and α-tubulin. (*P<0.05, **P<0.01; *t*-test).

DLC2 is a GAP and its depletion in non-synchronized cells indeed increased the GTP-bound RhoA and Cdc42 levels (Supplementary Fig. 3a). We next repeated the activity measurements with cells that had first been arrested in prometaphase with Nocodazole followed by washout of Nocodazole for different periods of time^32^. Arrested cells did not reveal any difference in the activity (Fig. 3a and Supplementary Fig. 3b). Forty-five minutes after Nocodazole washout, which corresponds to the metaphase peak, DLC2 depletion resulted in increased levels of Cdc42-GTP, but not of RhoA-GTP. Once mitotic cells reached telophase/cytokinesis (90 min), increased Cdc42-GTP levels had disappeared, possibly due to the activation of other mechanisms of Cdc42 regulation active during the end of mitosis. Hence, the depletion of DLC2 resulted in the upregulation of Cdc42 activity in mitotic cells correlating with the chromosome alignment defect. Localization of active Cdc42 in non-synchronized cultures further supported the conclusion that Cdc42 activation was enhanced in DLC2-depleted mitotic cells (Fig. 3b,c). To test an involvement of Cdc42 directly, we performed siRNA co-transfection experiments and observed that siRNAs targeting Cdc42 attenuated the chromosome alignment defect. Thus, Cdc42 is a functionally relevant substrate of DLC2 during mitosis (Fig. 3d,e).

### Kif1B is a mitotic kinesin and cooperates with DLC2

**Figure 4:**
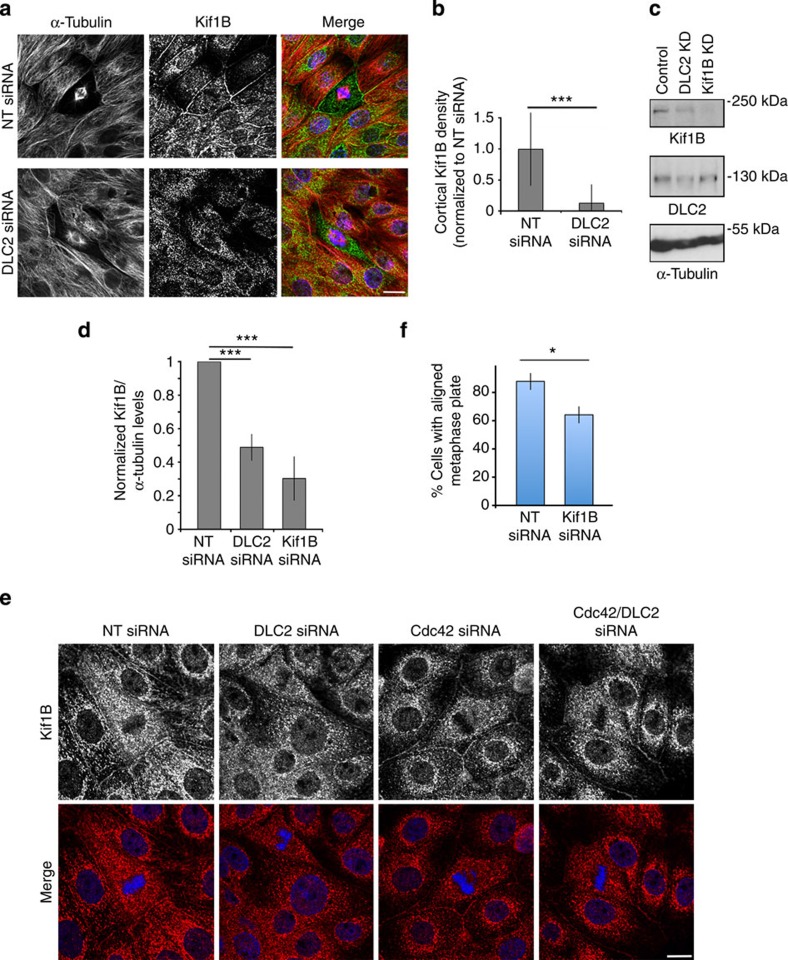
DLC2 regulates the cortical recruitment of Kif1B. (**a**) Control and DLC2-depleted HCE cells stained for α-tubulin (red), Kif1B (green) and DNA (blue). (**b**) Quantification of Kif1B staining at the lateral cell cortex (means±1 s.d.; *n*=10 cells). (**c**) Immunoblots of extracts from cells transfected with control, DLC2- and Kif1B-specific siRNAs. Note: the depletion of DLC2 also reduces the expression of Kif1B. (**d**) Immunoblots such as those shown in **c** were quantified by densitometry, and the density ratios of Kif1B divided by α-tubulin were calculated and normalized to control cells (shown are means±1 s.d.; *n*=3 experiments; at least 60 mitotic cells were counted per condition in each of these three experiments). (**e**) Control, DLC2-, Cdc42- and Cdc42/DLC2-depleted HCE cells were stained for DNA (blue) Kif1B (red). (**f**) Quantification of cells with bipolar spindles with aligned and misaligned metaphase plates transfected with control and Kif1B siRNAs (means±1 s.d.; *n*=3 individual experiments). Scale bars, 10 μm. (*P<0.05, ***P<0.001; *t*-test).

We next asked whether Kif1B not only interacts with DLC2 but participates in the regulation of mitosis. DLC2 depletion strongly reduced the cortical Kif1B staining; at least in part, this seemed to be due to a reduction in total Kif1B expression (Fig. 4a–d). Kif1B depletion did not affect DLC2 expression (Fig. 4d). The effect of DLC2 depletion on Kif1B localization could be rescued by the co-transfection of Cdc42 siRNAs, indicating that Kif1B localization is sensitive to active Cdc42 levels (Fig. 4e and Supplementary Fig. 4). Hence, Kif1B expression is not directly affected by DLC2 but requires correct control of Cdc42 activity.

Given the effect of DLC2 and Cdc42 on Kif1B localization, we asked whether the depletion of the kinesin also affected mitosis. This possibility was supported by the observation that depletion of the kinesin also led to increased Cdc42-GTP levels in interphase cells and during mitosis 45 min after the Nocodazole washout (Supplementary Fig. 3c,d). Indeed, Kif1B knockdown phenocopied the chromosome alignment defect of DLC2 depletion and also affected the junctional integrity of mitotic cells (Fig. 4f and Supplementary Fig. 5). These phenotypic data along with the complex formation suggest a role for Kif1B in mitosis and indicate that DLC2 and the kinesin cooperate.

### DLC2 and Kif1B regulate the cortical localization of mDia3

**Figure 5:**
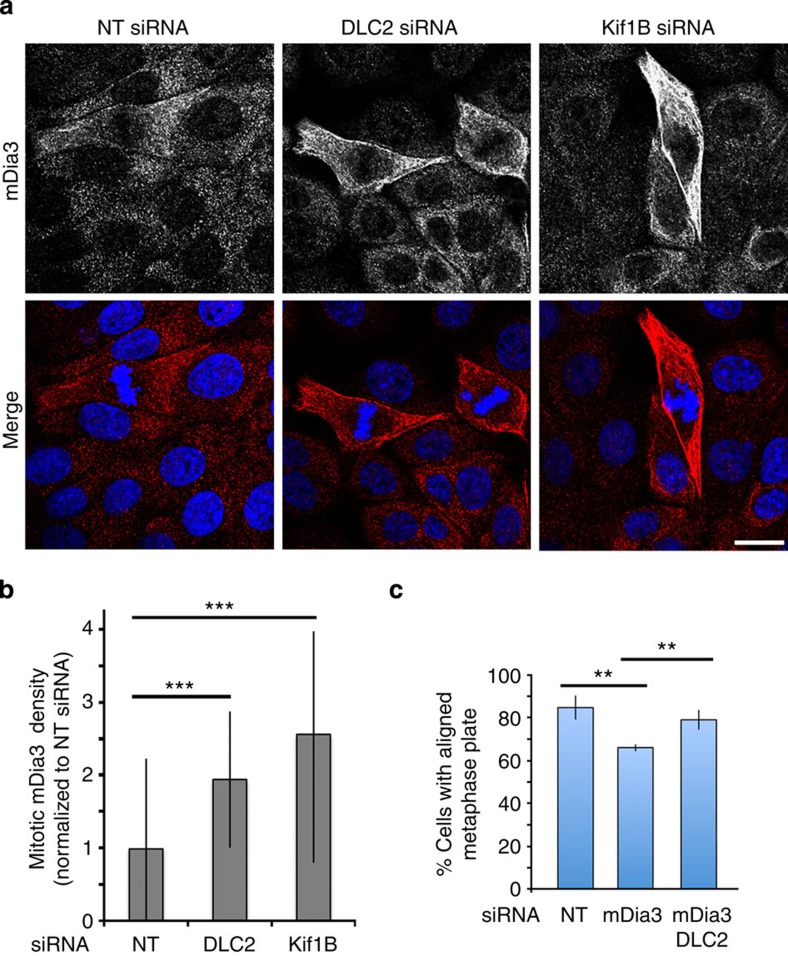
DLC2 and Kif1B regulate mDia3 recruitment at the cell cortex. (**a**,**b**) Control, DLC2- and Kif1B-depleted cells were stained for mDia3. **b** shows a quantification of mDia3 staining at the lateral cell cortex (means±1 s.d.; *n*=10 cells). Note: cortical mDia3 staining is elevated when Cdc42 activity is increased and cortical Kif1B levels are low (see Fig. 4). (**c**) Quantification of cells with bipolar spindles with aligned and misaligned metaphase plates transfected with the indicated siRNAs (means±1 s.d.; *n*=3; at least 60 mitotic cells were counted per condition in each of these three experiments). Scale bars, 10 μm. (**P<0.01, ***P<0.001; *t*-test).

We next asked which Cdc42 effector mechanism might get activated in DLC2-depleted cells. mDia3 is a Cdc42 effector required for mitosis and was suggested to regulate chromosome attachment to the spindle^33^,^34^. Hence, we tested whether depletion of DLC2 or Kif1B affected mDia3 localization. Indeed, the depletion of either protein increased cortical mDia3 staining (Fig. 5a,b). We could not detect clear staining for mDia3 on the metaphase plate, suggesting that a primary location is at the cell cortex in metaphase and interphase. If mDia3 was depleted, the number of cells with misaligned chromosomes increased, in agreement with the previous observations that mDia3 is required for normal mitosis (Fig. 5c and Supplementary Fig. 6a)^13^,^18^,^35^. Co-transfection of siRNAs targeting DLC2 and mDia3 partially rescued the defect in metaphase plate organization induced by the downregulation of individual proteins (Fig. 5c), indicating that mDia3 is a functionally relevant Cdc42 effector downstream of DLC2.

### DLC2 depletion delays metaphase and checkpoint inactivation

**Figure 6:**
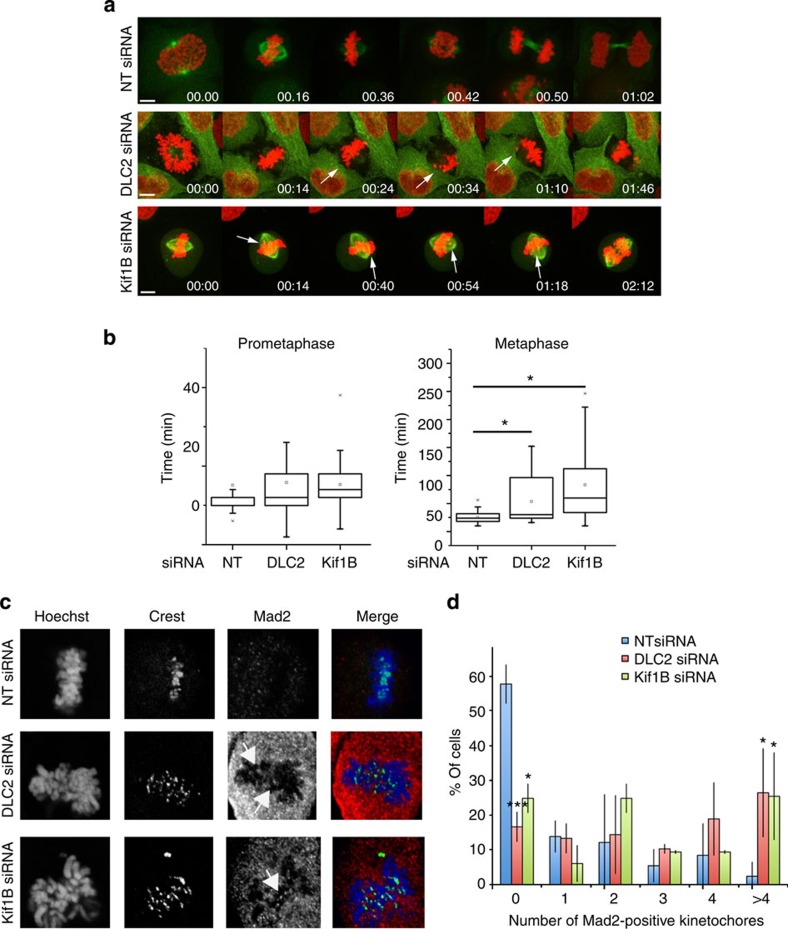
DLC2 and Kif1B are required for mitotic checkpoint satisfaction. (**a**) HeLa cells expressing H2B-mCherry and α-tubulin-GFP were transfected with control, DLC2 or Kif1B siRNAs and filmed through mitosis. Pictures were taken with a Spinning Disc confocal microscope every 2 min (see Supplementary Movies 1–3). (**b**) Length of prometaphase and metaphase were quantified (Box-and-whisker plots, *n*=30). (**c**) Examples of control, DLC2- and Kif1B siRNA-transfected HCE cells stained for CREST (green) and Mad2 (red). (**d**) Quantification of the percentage of cells showing a specific number of Mad2-positive dots at the kinetochores. DLC2 and Kif1B KD cells show higher percentages of cells with more than one Mad2-positive dot (shown are means±1 s.d.; *n*=3 experiments). Scale bars, 10 μm. (*P<0.05, ***P<0.001; analysis of variance).

To test whether DLC2 and Kif1B affect the formation or maintenance of the metaphase plate, we performed live imaging. We used HeLa cells as their mitotic behaviour is well established and employed a cell line expressing mCherry-H2B and GFP-α-tubulin. Depletion experiments showed that DLC2 and Kif1B were required for normal mitotic progression, as in HCE cells, regulating metaphase length (Fig. 6a,b, Supplementary Fig. 6b and Supplementary Movies 1–3). In depleted cells, the metaphase plate initially formed at normal rates, but, then, chromosomes often escaped from the plate, and the spindle exhibited excessive rocking and spinning.

We next asked if the metaphase delay was checkpoint dependent by staining for Mad2. Counting of Mad2-stained kinetochores confirmed that the depletion of either protein increased the number of unattached chromosomes in metaphase cells (Fig. 6c,d). Aurora B kinase is a major component of the checkpoint system, and it is activated by phosphorylation, when unattached kinetochores are present^36^. Depletion of DLC2 or Kif1B indeed prolonged Aurora B phosphorylation (Supplementary Fig. 6c). Similarly, the Aurora B substrate Dsn1 was more strongly phosphorylated in metaphase cells after DLC2 and Kif1B depletion, further supporting a defect in maintenance of chromosome alignment and kinetochore attachment (Supplementary Fig. 6d,e).

### DLC2 and Kif1B control spindle positioning

**Figure 7:**
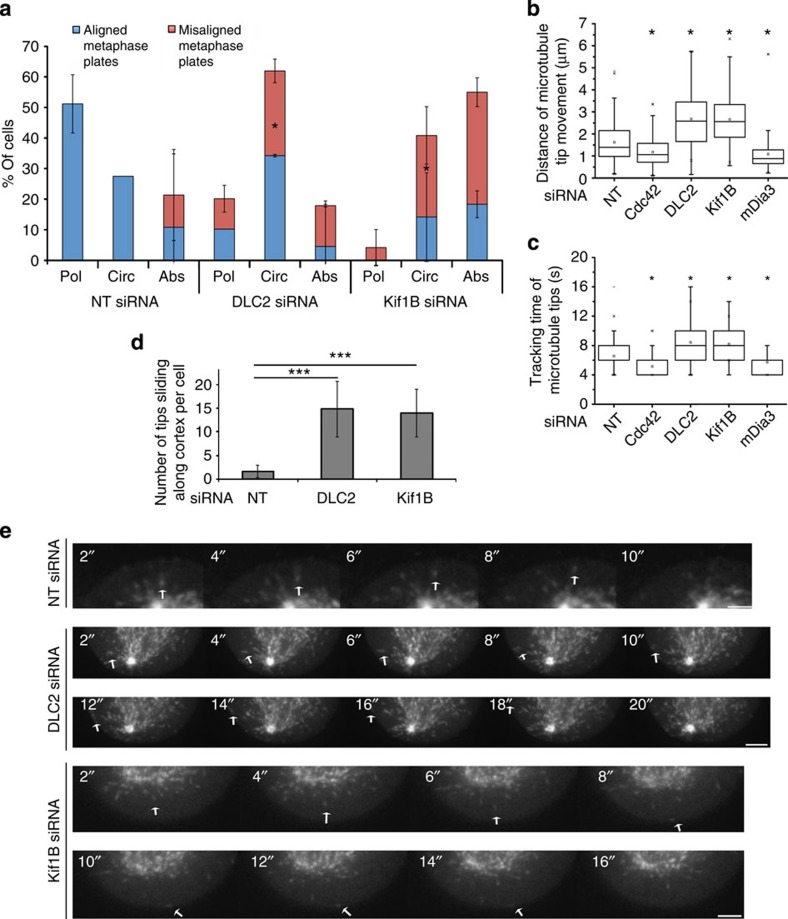
DLC2 and Kif1B regulate spindle positioning and microtubule growth. (**a**) Hela cells stably expressing mCherry-lifeact and GFP-α-tubulin were transfected with siRNAs and plated onto micropatterned dishes with parallel fibronectin lines. Time-lapse recordings were then made collecting images every minute to follow actin behaviour and spindle orientation (see Supplementary Movies 4–6 and Supplementary Fig. 7). The movies were then quantified by calculating the percentages of cells with a specific actin behaviour based on kymographs and spindle orientation (Pol, polarized actin; Circ, circular movement of actin; Abs, absent actin polarization). Blue bars represent cells with mitotic spindles aligned with the fibronectin line; red bars, misaligned spindles. Significance was calculated between corresponding subgroups (shown are means±1 s.d.; *n*=3 experiments). (**b**–**e**) HeLa cells stably transfected with GFP-EB3 and transfected with the indicated siRNAs were filmed every 2 s. Microtubule dynamics was then quantified in at least 10 cells, following 10 plus-ends per cell (see Supplementary Movies 7–9). The box-and-whisker plots show total distance tracked (**b**) and total time tracked (**c**) (*n*=45 microtubule tips). Microtubule tips sliding along the cell cortex were also quantified in 10 cells per type of siRNA transfection (**d**). The still images in panel **e**, taken every 2 s, show examples of microtubule tips tracked. Scale bars, 1 (upper panel in **e**) and 2 μm (lower panels in **e**). (*P<0.05, ***P<0.001; analysis of variance).

Because DLC2 depletion stimulated the cortical recruitment of mDia3, a Cdc42 effector that does not only stabilize microtubules but also nucleates actin polymerization, we speculated that DLC2 depletion interferes with the normal organization of actin and microtubule interactions; hence, DLC2-deficient cells might be defective in normal spindle positioning or orientation. To test for defects in spindle positioning, we plated HeLa cells stably expressing mCherry-lifeact, to monitor actin dynamics and GFP-α-tubulin, to follow spindle movements, on micropatterned substrates with regular fibronectin lines. This introduces an external constrain, resulting in an alignment of the spindle axis with the fibronectin line and the formation of an actin cap that is polarized along the fibronectin line or rotates (see Supplementary Fig. 7 for examples; Supplementary Movies 4–6)^37^. Depletion of DLC2 or Kif1B strongly affected both parameters. More than half the cells failed to align the spindle even though both types of cells still formed retraction fibres, indicating that they no longer responded to the external cues (Fig. 7a). Actin dynamics was altered as the percentage of cells with a rotating actin cap strongly increased. Spindle alignment and actin organization were not directly related, as spindle positioning was defective also in cells that still polarized subcortical actin, suggesting that the defect in spindle positioning was not simply due to the disorganized actin but to a defect in the spindle recognizing cortical cues. Determination of spindle angles relative to the substrate did not reveal defects in spindle orientation (Supplementary Fig. 8a,b). Measurements of pole-to-pole distances did also not reveal significant changes in response to DLC2 or Kif1B depletion (Supplementary Fig. 8c). Hence, the depletion of the two proteins affected neither the positioning of the poles relative to each other nor the orientation of the spindle.

### DLC2 and Kif1B control microtubule length

The mitotic spindle is thought to interact with the cell cortex via astral microtubules. Hence, we next asked whether DLC2 and Kif1B regulate microtubule dynamics. Analysis of microtubule growth by manual tracking of EB3-GFP-labelled plus ends of astral microtubules revealed that depletion of either protein resulted in microtubules growing for longer times and distances (Fig. 7b,c and Supplementary Fig. 9). Microtubule tips often seemed to glide along the cell cortex in DLC2-depleted cells, suggesting that the normal polymerization behaviour was disrupted (Supplementary Movies 7 and 8 and Fig. 7d,e). On the contrary, Cdc42 and mDia3 knockdown induced shorter microtubules growing for less time (Fig. 7b,c and Supplementary Movie 9). Hence, the interplay between DLC2/Kif1B and Cdc42/mDia3 regulates microtubule growth.

**Figure 8:**
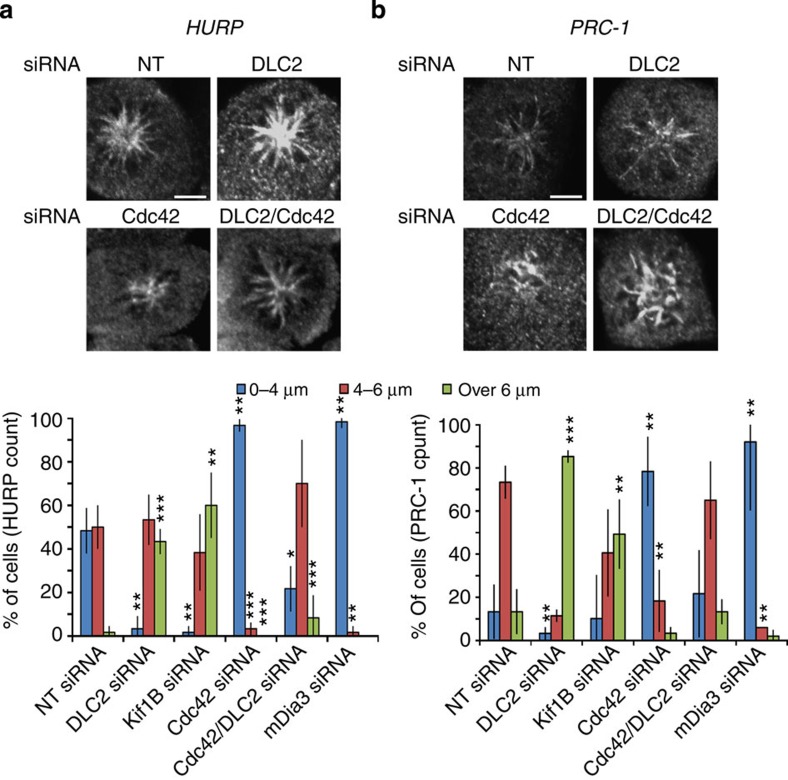
DLC2 and Kif1B regulate the formation of bipolar spindles after monastrol release. Confocal microscopy pictures for control, DLC2 and Kif1B, Cdc42, mDia3 and Cdc42/DLC2 siRNA-transfected HCE cells treated with monastrol. Cells were stained for HURP (**a**) and PCR-1 (**b**). The charts show the length of astral (HURP) and kinetochore (PCR-1) microtubules in HCE cells treated with monastrol (shown are means±1 s.d.; *n*=3 experiments). Scale bars, 10 μm. (*P<0.05, **P<0.01, ***P<0.001; *t*-test.)

**Figure 9:**
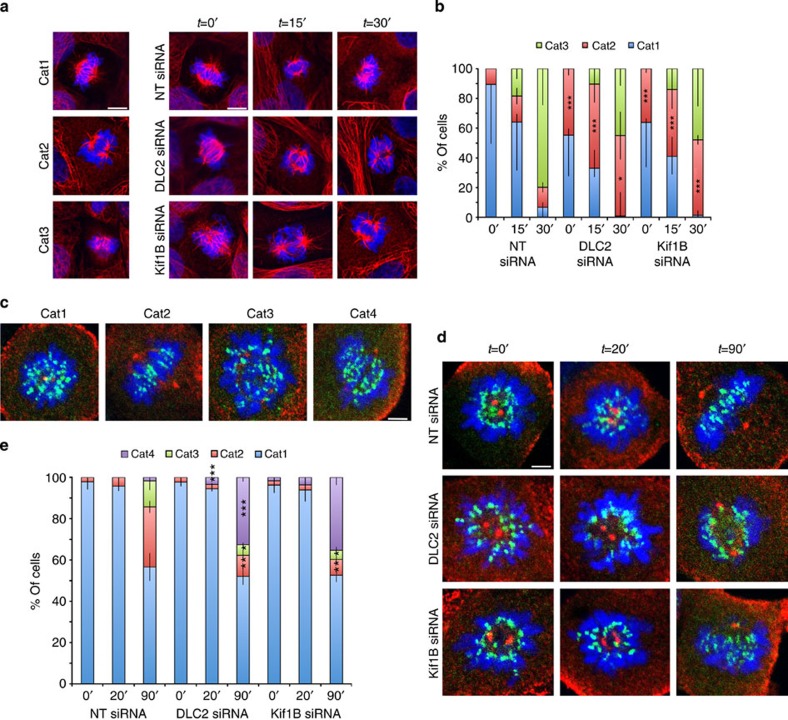
DLC2 and Kif1B regulate microtubule dynamics. (**a**) Cold treatment of HCE cells. After the cold treatment, HCE cells were fixed and stained for α-tubulin (red) and DNA (blue). Shown are the categories of cells counted in the chart in **b**. Category 1, cells with an intact spindle and aligned metaphase plate; category 2, cells with bipolar spindles containing some long fibres and misaligned metaphase plates; category 3, cells with severe disruption of spindles. Examples of images obtained with NT-, DLC2- and Kif1B siRNA-transfected cells in response to cold treatment are also shown. (**b**) Quantification of percentages of the three categories of microtubule staining through time for control, DLC2- and Kif1B siRNA-transfected HCE cells (shown are the means±1 s.d.; *n*=3 experiments). (**c**–**e**) Spindle reformation after monastrol washout. After different periods of monastrol washout, HCE cells were fixed and stained for γ-tubulin (red), the kinetochore marker CREST (green) and DNA (blue). The cells were then imaged and divided into categories (**c**) Category 1, cells with monopolar spindles with intact metaphase plates; category 2, bipolar spindles with intact metaphase plates; category 3, bipolar spindles with sever metaphase plate scattering; and category 4, bipolar spindles with misaligned metaphase plate but with chromosomes less scattered than category 3 cells. (**d**) Example images of control, DLC2- and Kif1B siRNA-transfected cells treated with monastrol after washout of the drug. (**e**) Quantification of the percentages of the three categories through time for control, Cdc42- and Kif1B siRNA-transfected HCE cells (shown are means±1 s.d.; *n*=3 experiments). Scale bars, 10 μm. (*P<0.05, **P<0.01, ***P<0.001; *t*-test).

We next induced monopolar spindles using monastrol and stained for HURP, a marker for kinetochore microtubules, or PRC-1, which associates with non-kinetochore microtubules. Figure 8 shows that DLC2- or Kif1B-depleted cells formed longer and Cdc42- or mDia3-depleted cells shorter microtubules. Co-transfection of siRNAs targeting Cdc42 and DLC2 complemented each other, further supporting the importance of Cdc42 downstream of DLC2. The effect on HURP-positive microtubules suggests that DLC2 might also affect kinetochore microtubules. The role of the DLC2/Kif1B signalling module was further supported by the microtubule response to cold treatment. DLC2- and Kif1B-depleted cells formed fibres that were more stable than in control cells (Fig. 9a,b; less category 1 cells). In Cdc42- and mDia3-depleted cells, cold-resistant microtubules were shorter, and DLC2 and Cdc42 complemented each other (Supplementary Fig. 10a–c). In monastrol washout experiments, less than 10% of DLC2- and Kif1B KD-depleted cells assembled bipolar spindles with properly oriented and aligned metaphase plates (category 2 cells), further corroborating the importance of DLC2 and Kif1B in regulating microtubule dynamics (Fig. 9c–e).

### DLC2 and Kif1B ensure even chromosome segregation

**Figure 10:**
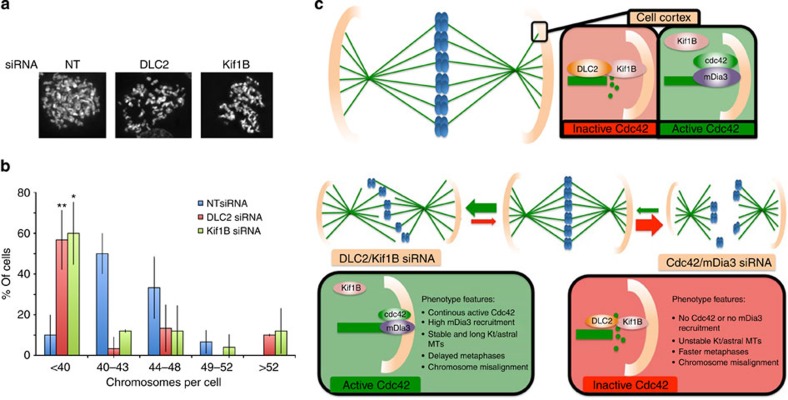
DLC2 and Kif1B are required for mitotic fidelity. (**a**,**b**) chromosome spreads of control, DLC2- and Kif1B siRNA-transfected HCE cells. **a** shows representative images and **b** shows a quantification of the percentage of cells with a particular chromosome number (shown are means±1 s.d.; *n*=3 experiments). Scale bars, 10 μm. (*P<0.05, **P<0.01; *t*-test). (**c**) Proposed model for the action of DLC2 and Kif1B in metaphase plate stabilization and spindle positioning. The cartoon shows that to position the spindle and maintain epithelial integrity balanced negative regulation of Cdc42 by DLC2 is required. If DLC2 is inactivated, Cdc42 activity is enhanced and leads to the activation of mDia3, triggering increased microtubule stability and disorganization of cortical actin. The increased microtubule length leads to a loss of normal tension control, distortion of the spindle and, hence, defects in metaphase plate maintenance and cohesion fatigue.

Chromosome attachment to the spindle is fundamental to ensure the equal partitioning of chromosomes between the two daughter cells and to prevent aneuploidy^38^,^39^. Therefore, we asked if DLC2 or Kif1B depletion affects chromosome partitioning as we had observed prolonged association of Mad2 with kinetochores. Figure 10a,b shows that most control siRNA-treated cells (>80%) had between 40 and 48 chromosomes. However, the majority of Kif1B and DLC2-depleted cells had fewer chromosomes (~60%) and 10–15% had more chromosomes. Hence, the tumour suppressor DLC2 and the kinesin Kif1B are required for chromosome stability.

## Discussion

Here we identified DLC2 and the kinesin Kif1B as key components of a regulatory network that guides microtubule dynamics during cell division by controlling the crosstalk between astral microtubules and the cortical actin cytoskeleton required for spindle positioning, mitotic progression and epithelial integrity (Fig. 10c). mDia3 is a critical effector of DLC2-regulated Cdc42 activity, regulating actin and microtubule organization and, thereby, spindle positioning and mitotic fidelity.

DLC2 and Kif1B are required for the maintenance of cell–cell junctions during mitosis and the stability of the metaphase plate. DLC2’s main substrate during mitosis is Cdc42, as reduced expression of Cdc42 could rescue the DLC2 phenotype. A Cdc42 GAP that is selectively required during mitosis, but not in interphase cells, for junction maintenance had thus far not been identified. MgcRacGAP/CYK-4 has been shown to regulate Cdc42 during mitosis by expressing a dominant negative mutant that led to a prometaphase arrest^40^. However, unlike DLC2, the depletion of MgcRacGAP/CYK-4 did not affect mitotic progression but caused a defect in cytokinesis^41^. The activity of Cdc42 peaks around metaphase and needs to be downregulated for mitotic progression^40^. Hence, DLC2 is required when Cdc42 activity starts to decline.

Cdc42 regulates spindle positioning as well as spindle orientation; however, DLC2 depletion only affected spindle positioning. Spindle positioning has thus far been reported to be caused by defects in Cdc42 activation (that is, the depletion of Cdc42 itself or of specific GEFs required for normal spindle orientation)^12^,^14^,^23^,^24^,^25^. Our data now indicate that enhanced Cdc42 activity may not directly affect spindle orientation but does disturb spindle positioning, suggesting that the two processes are differentially regulated and that only spindle positioning requires correct termination of Cdc42 signalling. This mechanistic difference is also supported by the activated mechanisms, as spindle orientation requires PAR protein signalling, whereas the spindle positioning defects induced by DLC2 depletion lead to enhanced microtubule length.

The kinesin Kif1B and DLC2 are mechanistically tightly linked. Kif1B had previously not been associated with mitosis but axonal transport, which is thought to be the reason why its deficiency causes Charcot–Marie–Tooth disease^42^. Kif1B depletion led to increased astral microtubule stability and deregulation of spindle positioning. As this reflects the DLC2 phenotype, it seems that the main function of Kif1B during mitosis is to cooperate with DLC2. Hence, Kif1B might play a role in DLC2 regulation. It is also possible that DLC2 regulates Kif1B, which then in turn plays a direct role in the regulation of microtubules, perhaps by functioning as a depolymerizing kinesin^43^.

The depletion of DLC2 and Kif1B affected chromosome attachment to kinetochore microtubules that compromises the maintenance of a metaphase plate. However, mitosis ultimately progressed but resulted in uneven chromosome segregation. One possibility is that chromosome misalignment in DLC2-depleted cells occurred due to cohesion fatigue followed by spindle checkpoint satisfaction^44^. Cdc42 has previously been suggested to regulate the attachment of kinetochores to microtubule plus ends, a process involving its effector mDia3 (refs 13, 18, 35, 44). It is possible that the attachment defect we observed in DLC2-depleted cells was caused by the deregulation of mDia3 at kinetochores. However, we were unable to detect clear mDia3 labelling of kinetochores despite the use of several mDia3 antibodies. As we detected mDia3 at the cell cortex and this cortical labelling was upregulated in response to DLC2 or Kif1B depletion, which leads to enhanced Cdc42 signalling, the cell cortex seems to be an important site of mDia3 function during mitosis.

Our data indicate that DLC2/Kif1B and Cdc42/mDia3 regulate the dynamics of microtubules in an opposing fashion: depletion of DLC2 or Kif1B induced the hyperstabilization of microtubules, whereas Cdc42 or mDia3 knockdown led to fibre instability. Both situations altered the mitotic spindle so that the spindle checkpoint satisfaction necessary for entry into anaphase was delayed to ensure that chromosomes are segregated correctly. The mitotic spindle structure is dynamic and continuously evolving; hence, the growth and shrinkage of microtubules must be carefully controlled so that the fibres have the right length^45^,^46^. We thus propose a model according to which DLC2 and Kif1B function as a tuner that regulates Cdc42 and, thereby, mDia3 activity and astral microtubule length (Fig. 9c). In the absence of DLC2/Kif1B, microtubules are hyperstable and, hence, become too long and generate a force pushing the spindle poles towards each other. As the distance between the poles remains constant, the metaphase plate becomes distorted and shows excessive spinning and defective positioning. At least in monopolar spindles, kinetochore microtubules behave like astral microtubules in response to DLC2/Kif1B inactivation, suggesting that the failure in metaphase plate maintenance might be further worsened by enhanced stability of kinetochore fibres, which also leads to attachment defects^47^,^48^,^49^. The DLC2 mechanism also affects cortical actin polarization in line with mDia3’s activity in actin organization. Hence, DLC2/Kif1B signalling coordinates mitotic spindle positioning with cell shape changes and maintenance of cell–cell adhesion.

DLC2 and Kif1B co-precipitate with p120 catenin, an adherens junction protein that has previously been linked to interacting with microtubules in interphase cells^50^,^51^. Different mechanisms have been suggested to link p120 catenin to microtubules, involving either a classical kinesin or a kinesin-14, a minus-end directed motor that associates with a protein complex that binds microtubule minus ends^50^,^51^. However, dynamic microtubule plus-ends have also been suggested to regulate adherens junction formation^52^. Hence, microtubule plus- and minus-end binding proteins regulate cell–cell adhesion in interphase cells. Currently, we do not know whether Kif1B binds to core adherens junction proteins directly; however, our data suggest that during mitosis interplay between microtubule plus-ends and adherens junction proteins regulates cell–cell adhesion.

DLC2 was originally identified as a tumour suppressor in hepatocytes and other epithelial cell types^27^. Kif1B depletion/inactivation has been linked to hepatocellular carcinomas as well as neuroblastomas^53^. Our data now indicate that the DLC2/Kif1B module is required for mitotic fidelity and the maintenance of stable chromosome numbers. Chromosome instability is a major driver of tumorigenesis; hence, it seems likely that maintenance of chromosome numbers is a pathologically relevant function of the DLC2/Kif1B module. DLC2 overexpression was reported to lead to a block in G1/S-phase progression due to repression of RhoA activity^26^. We also observed that transfection of DLC2 inhibited proliferation. Hence, DLC2 seems to function as a cell cycle regulator at different phases: G1/S-phase progression by controlling RhoA signalling and metaphase to anaphase progression by inhibiting Cdc42/mDia3 signalling.

DLC2 prolonged mitosis in two distinct human model cell lines. A recent report further reported that DLC2 depletion in a breast cancer cell line also results in an increased pool of cells in G2/M phase, without further investigating the underlying mechanism^54^. In mice, however, DLC2 is not required for development and did not aggravate diethylnitrosamine-induced hepatocarcinogenesis^55^. As the homologous proteins DLC1 and DLC3 have the same domain structure and have been reported to associate with cell–cell junctions, it is possible that these proteins might be able to at least partially compensate for each other in constitutive loss-of-function experiments such as the reported knockout mice^56^,^57^. However, it can also not be excluded that the relative importance of different DLC family members is different in different species. Moreover, based on the observations reported here, one would not expect that chemically induced tumours occur more frequently but that rates of spontaneous tumours increase in tissues that are renewed.

In conclusion, our results reveal that a signalling module formed by DLC2 and the kinesin Kif1B tunes mitotic Cdc42/mDia3 activity to guide microtubule growth and stability, actin dynamics and, thereby, astral microtubule crosstalk with the actin cytoskeleton. This regulatory network coordinates cell junction maintenance and planar spindle positioning, and ensures mitotic fidelity and tissue integrity.

## Methods

### Cell culture

HCEs were a gift from Min S. Chang (Vanderblit, University, Nashville, Tennessee)^58^. HeLa cells expressing H2B-GFP/α-tubulin-mRFP or EB3–tdTomato/eGFP–CENP-A were a gift from Patrick Meraldi (University of Geneva, Switzerland). HeLa and HCE cells were cultured in DMEM containing 10% heat-inactivated fetal bovine serum (FBS) and 100 μg ml^−1^ penicillin/streptomycin at 37 °C in a 5% CO2 atmosphere. For transient transfection, Lipofectime 2000 (Invitrogen) or JetPEI (Polyplus transfection) were used. Where indicated, cells were synchronized in prometaphase by incubation with Nocodazole 100 μM for 6 h (refs 32, 59). Nocodazole was washed off and cells were harvested or fixed and stained at the following time points: 45′ for the metaphase/anaphase peak or 90′ for the telophase/cytokinesis peak.

### DLC2 and Kif1B constructs

Human DLC2 and Kif1B constructs were generated based on the NCBI reference sequences NM_178006.3 for DLC2 and NM_015074.3 for Kif1B. For DLC2, fragments corresponding to the SAM domain (residues 1–130), the SAM domain together with the serine-rich domain (residues 1–600), the GAP domain (residues 631–888) and the STARD domain (residues 888–1113) were cloned into either pEGFP-C1 or pGEX4T3. For Kif1B, fragments corresponding to the motor domain (residues 1–366), the domain containing the FHA (residues 367–999), the intermediate domain (ID, residues 1,000–1,500), and the amino-terminal domain (NTD, residues 1501–1770) were cloned into either pEGFP-C1 or pRSET-A.

### Immunostaining and confocal microscopy

Hela cells were grown for 5 days on glass cover slides and HCE cells for 4 days in 48-well plates. On the last day, cells were fixed with cold methanol (−20 °C) for 10 min at −20 °C. The cells were then rehydrated with PBS for 5 min and blocked with PBS (0.5%), bovine serum albumin (BSA) (0.1%) and NaN3 for 15 min at room temperature. For actin staining, cells were fixed with 3% paraformaldehyde (PFA) in PBS, pH 7.4, for 20 min at room temperature, permeabilized with 0.5% Triton in PBS–BSA for 5 min and then incubated with PBS containing 0.5% BSA and 20 mM Glycine for 5 min. For microtubules staining, cells were prefixed at 37 °C for 15 min with 4% PFA diluted in a microtubules stabilization buffer (80 mM Pipes-NaOH pH 6.8/1 mM MgCl2, 1 mM EGTA and 0.1% Triton). They were then left at −20 °C in methanol for 3 min. Methanol was discarded, cells rehydrated with PBS for 5 min, and blocked with PBS (0.5%), BSA (0.1%) and NaN3 for 15 min at room temperature. HCE cells transiently transfected with DLC2 and Kif1B domain constructs were fixed in either methanol (5 min at −20 °C) or 3% PFA as described for Hela cells. The following primary antibodies were used: α-tubulin (Abcam—ab18251, rabbit, 1:200); β-catenin (Santa Cruz—sc31001, goat, 1:200); E-cadherin (BD Bioscience—610182, mouse, 1:1,000); Occludin (Invitrogen—33-1500, mouse, 1:1,000); p120-catenin (BD Bioscience—610134, mouse, 1:250); ZO-1 (1:200) (ref. 22); Kif1B (Bethyl Laboratories—A301-056A, Inc., rabbit, 1:200); DLC2 (Santa Cruz—sc67843, goat, 1:200; Santa Cruz—sc377054, mouse, 1:100); α-catenin (Sigma—C2081, rabbit, 1:200); GFP (Sigma—G1546, mouse, 1:200); HURP (Santa Cruz—sc68539 goat, 1:200); PRC-1 (Santa Cruz—sc8356, rabbit, 1:200); mDia3 (ECM Bioscience—DP4511, rabbit, 1:200); CREST (Sera Lab—AI-15-235-F, 1:1,000); APC (SantaCruz—sc7930, rabbit, 1:200); phospho-S100hDsn1 (kindly provided by Iain Cheeseman); Cdc42-GTP (NewEast Biosciences—26905, mouse, 1:100). Secondary antibodies conjugated to FITC, Cy3 or Cy5 that had been generated in Donkey and cross-absorbed (Jackson ImmunoResearch, Inc.) were reconstituted as instructed by the provider, brought to 50% glycerol and then used at a dilution of 1:300. For staining of F-actin, fluorescently labelled Phalloidin was mixed with the secondary antibodies, and for DNA detection Hoechst 33258 was used^21^. For confocal microscopy, a Leica TCS SPE confocal microscope with a 63x/NA1.4 objective and Leica Application Suite (LAS) software were used. Pictures were processed with Adobe Photoshop. Epifluorescence images were acquired with a Nikon Eclipse Ti-E inverted microscope using an Apochromat × 60,1.4 NA oil immersion objective, a CoolSNAP HQ2 camera (Photometrics), and Nikon software.

### Fusion protein production and pull downs

The DLC2 and Kif1B constructs were purified from BL21pLysS bacterial cultures that had been induced at 30 °C for 1 h with 1 mM isopropyl-β-D-thiogalactoside. For lysis, the bacteria were resuspended in 15 ml Lysis Buffer (PBS+0.5% Triton, 1 mM DTT (dithiothreitol)). Bacterial extracts were sonicated, centrifuged at 15,000*g* for 15 min and then stored at −80 °C (ref. 21). Equal amounts of GST or GST-fusion proteins were then loaded onto glutathione-agarose beads and, after washing, incubated with mammalian cell extracts or His-Kif1B recombinant proteins. After 2 h, the beads were washed with lysis buffer and PBS before analysis by SDS-polyacrylamide gel electrophoresis (SDS–PAGE) and immunoblotting.

### Tubulin sedimentation assay

Tubulin sedimentation assays were performed using the ‘Microtubule-binding protein spin-down assay’ kit from Cytoskeleton Inc. following the manufacturer’s protocol using GST-fusion proteins affinity purified with glutathione-agarose. Purified tubulin was polymerized and stabilized using taxol, and was then incubated at a concentration of 0.45 mg ml^−1^ with purified DLC2-GST constructs (5–20 μg ml^−1^) for 30 min at room temperature before centrifugation at 55,000 r.p.m. in a S100-AT3 Fixed angle rotor (Thermo Scientific) for 40 min at 25 °C. The fusion proteins had been diluted in the microtubule-binding buffer and had been centrifuged before the binding assays at 55,000 r.p.m. in the S100-AT3 rotor to remove aggregates. Solubility of the fusion proteins was then tested by centrifugation of samples processed in the same way as for the binding studies but without α-tubulin. Supernatant and pellet fractions were run on a 12% SDS–PAGE gel and then immunoblotted for GST and α-tubulin.

### Microcontact printing and cell patterning

Micropatterned dishes were kindly provided by Matthieu Piel. The micropatterned dishes used had parallel fibronectin lines (12 mm wide) printed. Cells were trypsinized and resuspended in fresh medium at a density of 10^4^ cells ml^−1^ before seeding onto the micropatterned coverslips. After 30 min of seeding, HeLa cells had attached to fibronectin. Non-adherent cells and debris were removed by replacing the medium with fresh medium.

### Drug treatments

To stabilize microtubules, Taxol 0.5 μM was incubated with the cells overnight at 37 °C. The MPS-1 inhibitor was used at 200 μM and was added to the cells at 37 °C right before filming. To induce the formation of monopolar spindles, cells were treated with monastrol 100 μm (Sigma-Aldrich) for 2 h at 37 °C (ref. 60). The last hour, MG132 was added. Cells were then washed three times with fresh medium to wash out the monastrol and bipolar spindle formation was analysed after 0, 20, 60 and 90 min. Over 60 cells were analysed per condition^61^,^62^.

### siRNA transfection

For siRNA transfections, INTERFERin (Polyplus transfection) was used as described^5^. The cells were incubated for a total of 96 h before analysis after adding the transfection mix, replacing the medium after 24 h. The following siRNA sequences were used: DLC2: 5′- GAUGU GAACUUCCAAAGGA -3′, 5′- CCAAGGCACUUUCUAUUGA -3′ and 5′- GGGCAACUUUCCACACUUA -3′; Kif1B: 5′- CGGAUAUCAACUACGAUGA -3′, 5′- GGGUAAUUUGCGUGUGCGU -3′ and 5′- CACAUUAAAGAAGAGAGCAUU -3′; mDia3: 5′- GAUCAGAGAUACUAAAUCA -3′, 5′- GAAGAUACUCAACGAAUUA -3′, 5′- CGAUUUAACUCAUCUGAUA -3′ and 5′- GAAUUACGAUCGGGUAUAU -3′; Cdc42: 5′- CGGAAUAUGUACCGACUGU -3′ and 5′- GAUGACCCCUCUACUAUUG -3′; RhoA: 5′- AUGGAAAGCAGGUAGAGUU -3′, 5′- GAACUAUGUGGCAGAUAUC -3′, 5′- GAAAGACAUGCUUGCUCAU -3′ and 5′- GAGAUAUGGCAAACAGGAU -3′. Control siRNAs were non-targeting siRNAs from Sigma.

### K-fibre stability

The cells were put on ice and then fixed after 0, 10, 15, 20, 25 and 30 min. To compare the Control and knockdown of DLC2, Kif1B, Cdc42 and mDia3, cells were arranged in classes. For the experiment that regards the control and knockdown of DLC2 and Kif1B cells, the three classes for quantification purposes were regular mitotic spindles, spindles with short K-fibres and spindles with long K-fibres. On the contrary, for the experiment with control and knockdown of Cdc42 and mDia3, the three classes were regular mitotic spindles, spindles with short K-fibres, short fibres with defects in chromosome alignments^63^.

### Chromosome spread

Chromosome spreads were performed by treating HCE cells for 2 h with Nocodazole (3.3 μM) at 37 °C and then for 20 min in 75 mM KCl Nocodazole (3.3 μM) at 37 °C to induce a hypotonic shock. Cells were rinsed with PBS and then fixed first with methanol at −20 °C for 3 min and then 4% PFA in PBS for 20 min at room temperature. Cells were blocked with PBS containing 0.01% Triton-X-100 and 10% FBS. To quantify the number of chromosomes present in chromosome spreads, CREST was used to stain the kinetochores. Kinetochore pairs were counted in a minimum of 50 cells from each condition^60^,^64^,^65^.

### Western blotting

Protein lysates were prepared for SDS–PAGE analysis by washing cells twice with PBS and then adding sample buffer with urea (2% SDS, 10% glycerol, 0.001% bromophenolblue, 0.1 M DTT, 0.0625 M Tris/pH 6.8 and 6 M urea). After homogenizing three times with a 23-gauge microneedle, samples were heated at 100 °C for 10 min. The following primary antibodies were used to detect proteins: α-tubulin (mouse, 1:20) (ref. 66), E-cadherin (BD Biosciences—610182, mouse, 1:1,000); p120-catenin (BD Biosciences—610134, mouse, 1:1,000); Kif1B (Bethyl Laboratories—A301-056A, Inc., rabbit, 1:1,000); DLC2 (Santa Cruz—sc67843, goat, 1:200); mDia3 (ECM Bioscience—DP4511, rabbit, 1:1,000); phospho-Aurora B (T232) (Abcam—ab61074, rabbit, 1:200); Cdc42 (Santa Cruz—sc89, mouse, 1:100). Uncropped versions of all blots are found in Supplementary Fig. 11.

### Immunoprecipitations

Cells were plated in a 14-cm plate and when they reached confluence, they were extracted at 4 °C using 2 ml of 0.5% Triton-X-100 in PBS. A cocktail of protease inhibitors (10 μg ml^−1^ leupeptin, 10 μg ml^−1^ aprotinin, 10 μg ml^−1^ pepstatin A, 50 μg ml^−1^ benzamide and 1 mM PMSF (phenylmethyl sulphonyl fluoride)) and phosphotase inhibitors (10 mM sodium fluoride, 10 mM sodium pyrophosphate and 4 mM sodium orthovanadate) was added to the lysis buffer before extraction. The samples were centrifuged for 2 min at 4 °C. To reduce non-specific binding, 100 μl of inactive sepharose beads were incubated with the resulting supernatants for 30 min. Samples were spun again at 7,000 r.p.m. for 2 min and the supernatants were transferred to 2 μg of antibody conjugated to Protein G-sepharose beads. Negative control beads were prepared using unrelated IgGs of the same species. After incubating at 4 °C for 2 h, the beads were washed twice with 1 ml of 0.5% Triton-X-100 in PBS and once with PBS. 60 μl SDS–PAGE sample buffer with urea was then added and the samples were boiled for 10 min at 100 °C.

### G-LISA RhoGTPase activation assay

Cells were transfected with the appropriate siRNAs in 12-well plates and analysed for levels of active RhoA, Cdc42 and Rac1 using the respective G-LISA assay kits from Cytoskeleton Inc.^21^ Protein concentration of each extract was measured to load equal amounts of samples in the G-LISA assays. Protein concentrations and horseradish peroxidase assays were measured using the protocols and reagents provided in the kit and using a FLUOstar OPTIMA microplate reader (BMGLabTech, Offenburg, Germany).

### Time-lapse microscopy

Cells were plated into 35-mm glass bottom dishes (14 mm, No. 1.5 coverglass; MatTek Corporation) and transfected with siRNAs. The medium was replaced at least 6 h before filming with L-15/10% FBS. Four-dimensional data sets were acquired with a spinning disc confocal system (Yokogawa) equipped with an electron multiplying charge-coupled device camera (iXonEM+; Andor) and a CSU-22 unit (Yokogawa) based on an inverted microscope (TE2000-U; Nikon). Two laser lines (488 and 561 nm) were used for near-simultaneous excitation of GFP and mRFP, and the system was driven by NIS Elements 3.0 software (Nikon). Time-lapse imaging of z stacks with 0.7-μm steps covering the entire volume of the mitotic apparatus were collected every 1–2 min (Supplementary Movies 1–6) or every 2 s (Supplementary Movies 7–9) with a plan-apochromat 1.40 NA × 60 immersion oil objective^3^. Brightfield microscopy was performed on a Nikon Eclipse TE2000-U microscope driven by NIS Elements 3.0 software with × 63 or × 100 objectives. Time lapse was performed every 30 s, 1 or 2 min, depending on the type of experiment.

### Statistical analysis and data presentation

Quantification of spindle angles and length was performed using ImageJ. Images were collected by scanning every 0.01 μm the entire volume of the mitotic spindle, for the angle measurements, and every 0.5 μm for the length analysis. Mitotic spindle length was calculated as the distance between the two spindle poles. The angle between the spindle axis and the substrate was defined as the angle between a line parallel to the substrate and the line crossing the two spindle poles. Fluorescent labelling intensity analysis was performed by selecting areas of interests in the images and the density was determined; 10 images were analysed per condition and label. Background was subtracted and corrected values were compared among control and knockdown conditions. For aurora B substrates, the maximum value in the control was identified and used as a threshold to define how many kinetochores show higher intensity in the knockdown cells. For comet tracking, GFP-EB3 stably transfected HeLa cells were imaged every 2 min. Individual comets were manually tracked using the ‘Manual Tracking’ plugin of ImageJ. Comets were followed continuously until the fluorescent signal disappeared. Three parameters were extracted from the tracking: comet total speed, total distance and total time. The comet time was defined as the continuous time a single comet track was followed (in seconds). The comet distance as the distance the comet travelled until the signal disappeared (μm). The comet speed by the formula *v*=mean distance (μm)/time (s) and it represents the mean velocity a comet travels. In charts, where the data are represented as box-whisker plots, the box size represents 75% of the population and the line inside the box represents the median of the sample. Maximum (in the upper quartile) and the minimum (in the lower quartile) values are represented by the size of the bars (whiskers). A parametric one-way analysis of variance was used to perform the statistical analysis for multiple groups. These statistical analyses were performed using SigmaStat 3.5 (Systat Software, Inc.). In the case that only two experimental groups were compared, *t*-tests were performed. Bar graph represent means±1 s.d., and the indicated numbers of experiments refer to independent experiments used to calculate the presented data.

## Author contributions

E.V. designed and performed the experiments. For the live imaging experiments, E.V. was supported and advised by J.G.F. and H.M. The project was devised by E.V., M.S.B. and K.M. All authors contributed to writing the manuscript.

## Additional information

**How to cite this article:** Vitiello, E. *et al*. The tumour suppressor DLC2 ensures mitotic fidelity by coordinating spindle positioning and cell–cell adhesion. *Nat. Commun.* 5:5826 doi: 10.1038/ncomms6826 (2014).

## Supplementary Material

Supplementary Figures Supplementary Figures 1-11

Supplementary Movie 1 HeLa cells expressing H2B-mCherry and α-tubulin-GFP were transfected with control siRNAs and filmed through mitosis. Pictures were taken with a Spinning Disc confocal microscope every 2 minutes.

Supplementary Movie 2 Description: HeLa cells expressing H2B-mCherry and α-tubulin-GFP were transfected with DLC2 siRNAs and filmed through mitosis. Pictures were taken with a Spinning Disc confocal microscope every 2 minutes.

Supplementary Movie 3 HeLa cells expressing H2B-mCherry and α-tubulin-GFP were transfected with Kif1B siRNAs and filmed through mitosis. Pictures were taken with a Spinning Disc confocal microscope every 2 minutes.

Supplementary Movie 4 HeLa cells stably transfected with m-Cherry-lifeact and GFP-EB3 were plated onto micropatterned dishes and filmed every minute to follow actin behaviour in mitosis. Shown is an example of cells with polarized actin dynamics.

Supplementary Movie 5 HeLa cells stably transfected with m-Cherry-lifeact and GFP-EB3 were plated onto micropatterned dishes and filmed every minute to follow actin behaviour in mitosis. Shown is an example of cells with circular actin dynamics.

Supplementary Movie 6 HeLa cells stably transfected with m-Cherry-lifeact and GFP-EB3 were plated onto micropatterned dishes and filmed every minute to follow actin behaviour in mitosis. Shown is an example of cells absent actin dynamics.

Supplementary Movie 7 HeLa cells stably expressing GFP-EB3 were transfected with control siRNAs. Images were then collected every 2 seconds to follow microtubule tips.

Supplementary Movie 8 HeLa cells stably expressing GFP-EB3 were transfected with Dlc2 siRNAs. Images were the collected every 2 seconds to follow microtubule tips. Note, microtubule tips often seemed to glide along the cell cortex, indicating that the normal polymerization behaviour was disrupted.

Supplementary Movie 9 HeLa cells stably expressing GFP-EB3 were transfected with control Cdc42 siRNAs. Images were then collected every 2 seconds to follow microtubule tips.
