# Supplementary material for: The tumour suppressor DLC2 ensures mitotic fidelity by coordinating spindle positioning and cell–cell adhesion
Source: Nat Commun. 2014 Dec 18;5:5826. doi: 10.1038/ncomms6826 (PMC4284802; doi:10.1038/ncomms6826)
Supplement: Supplementary Figures — 1-11 [file ncomms6826-s1.pdf]

SUPPLEMENTARY FIGURES

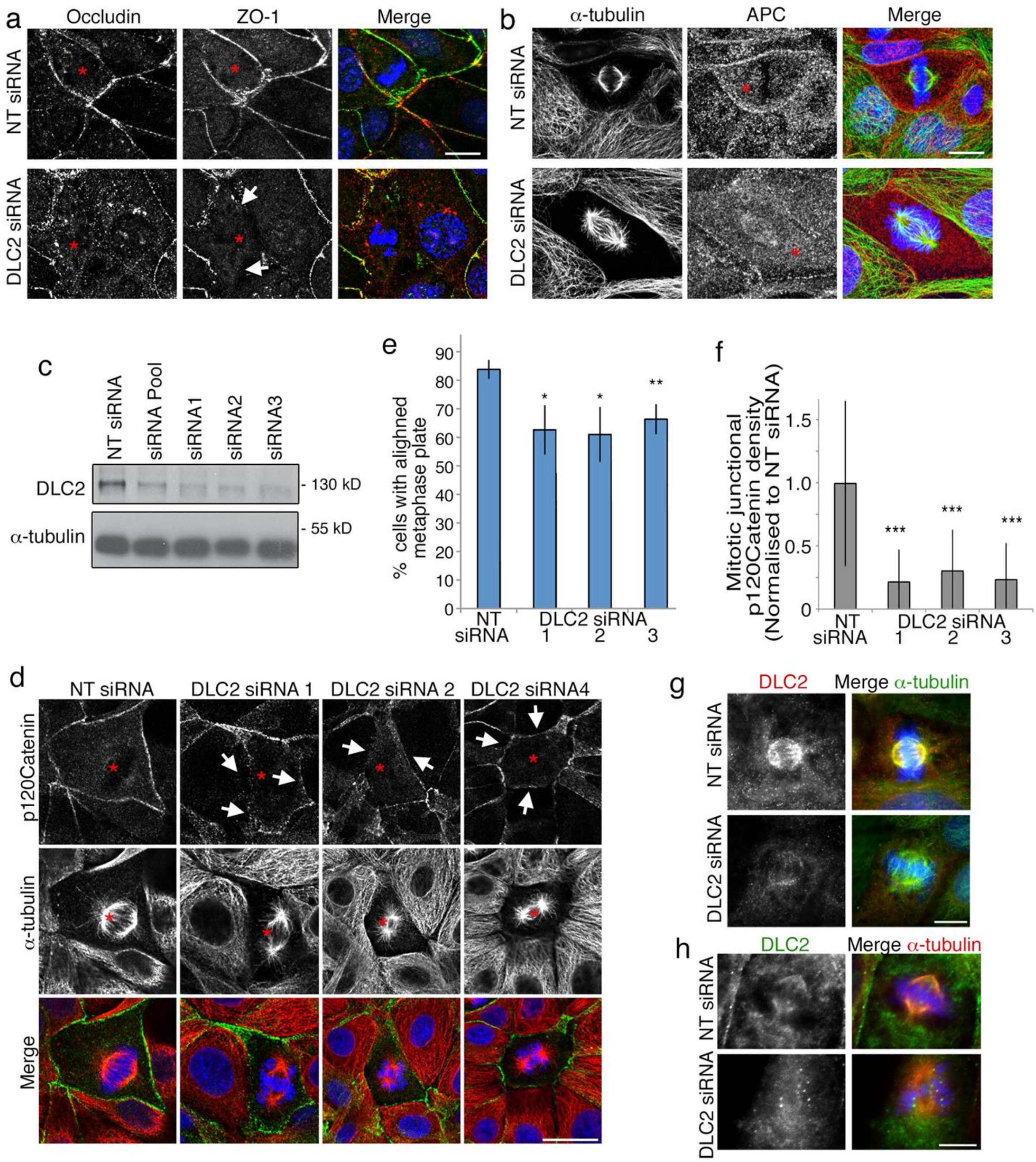

Supplementary Figure 1 DLC2 depletion affects junction maintenance and spindle stability. (**a**) Control and DLC2 depleted HCE cells stained for the tight junction proteins Occludin (green), ZO-1 (red) and DNA. (**b**) Control and DLC2 depleted HCE cells stained for APC (red), α-tubulin (green), and DNA (blue). (**c**) Immunoblot for DLC2 of total extracts of cells transfected with control and DLC2 siRNAs. α-tubulin was used as a loading control. (**d**) Control and DLC2 siRNA transfected HCE cells stained for the adherens junction protein p120Catenin (green), α-tubulin (red), and DNA (blue). (**e**, **f**) Quantification of cells with bipolar spindles with aligned and misaligned metaphase plates (**e**) and junctional p120Catenin intensity (**f**) in control and DLC2 siRNA transfected cells were quantified in images such as those shown in panel d (shown are means ± 1 SD; * p<0.05, ** p<0.01, *** p<0.001, t-test; n=3 experiments). (**g, h**) Control and DLC2 siRNA transfected HCE cells stained for DLC2 (red) and α-tubulin (green). Goat (g) and mouse (h) anti-DLC2 antibodies were used. Asterisks label mitotic cells and arrows point to dissociated cell-cell junctions. Scale bars, 10 μm.

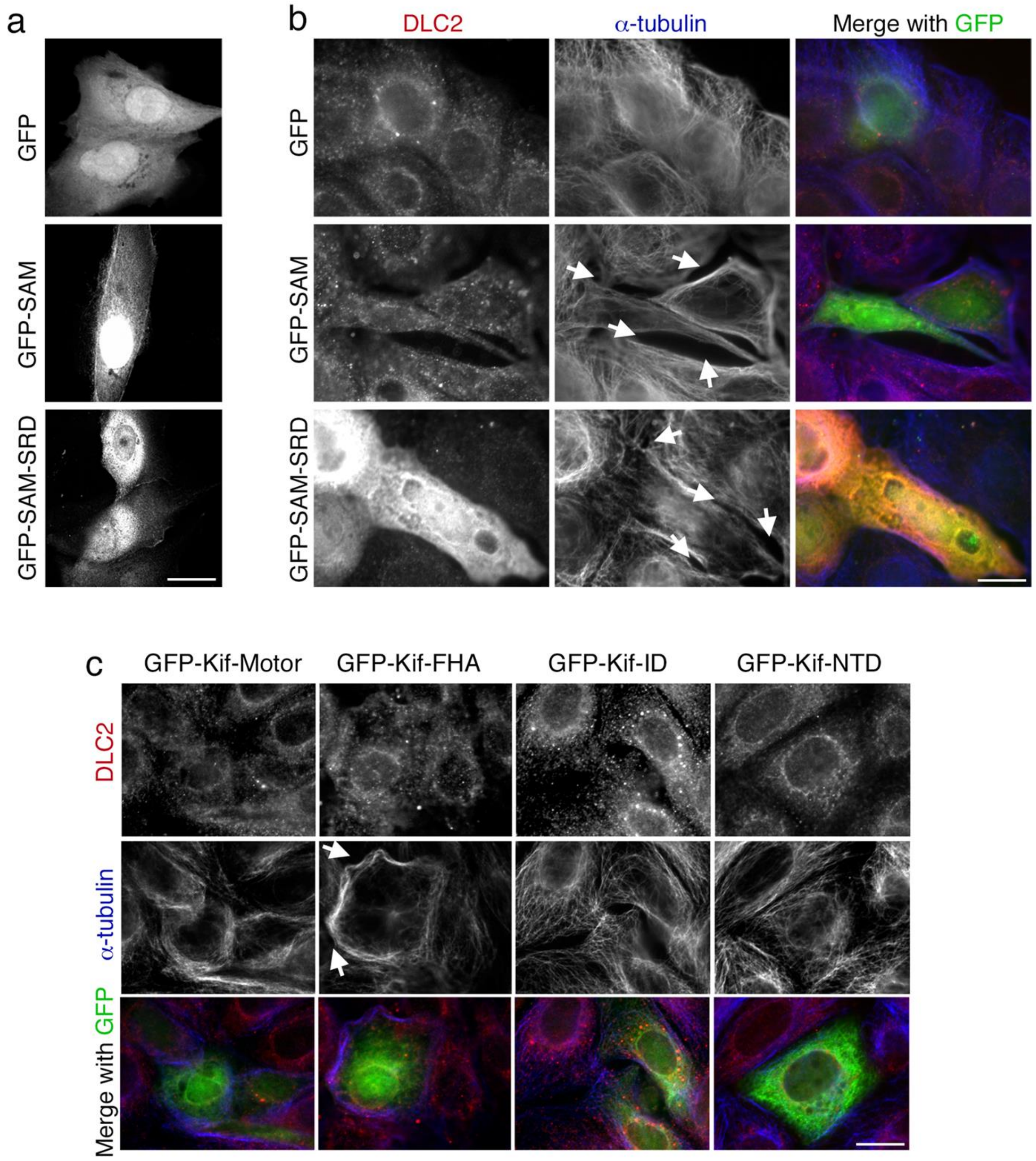

Supplementary Figure 2 Expression of GFP-tagged domain constructs in HCE cells. The indicated GFP-tagged domain constructs were transiently transfected into HCE cells. (**a**) Cells expressing relatively low amounts of GFP-tagged proteins containing the SAM domain show a partial association with cell-cell contacts. (**b**) In cells expressing high amounts of the SAM domain constructs, microtubules appear more peripheral and cells loose cell-cell contact (gaps between cells are labelled with arrows). (**c**) HCE cells expressing Kif1B domain constructs. Only the FHA domain construct, which binds DLC2, induces a redistribution of microtubules. Scale bars, 10 µm.

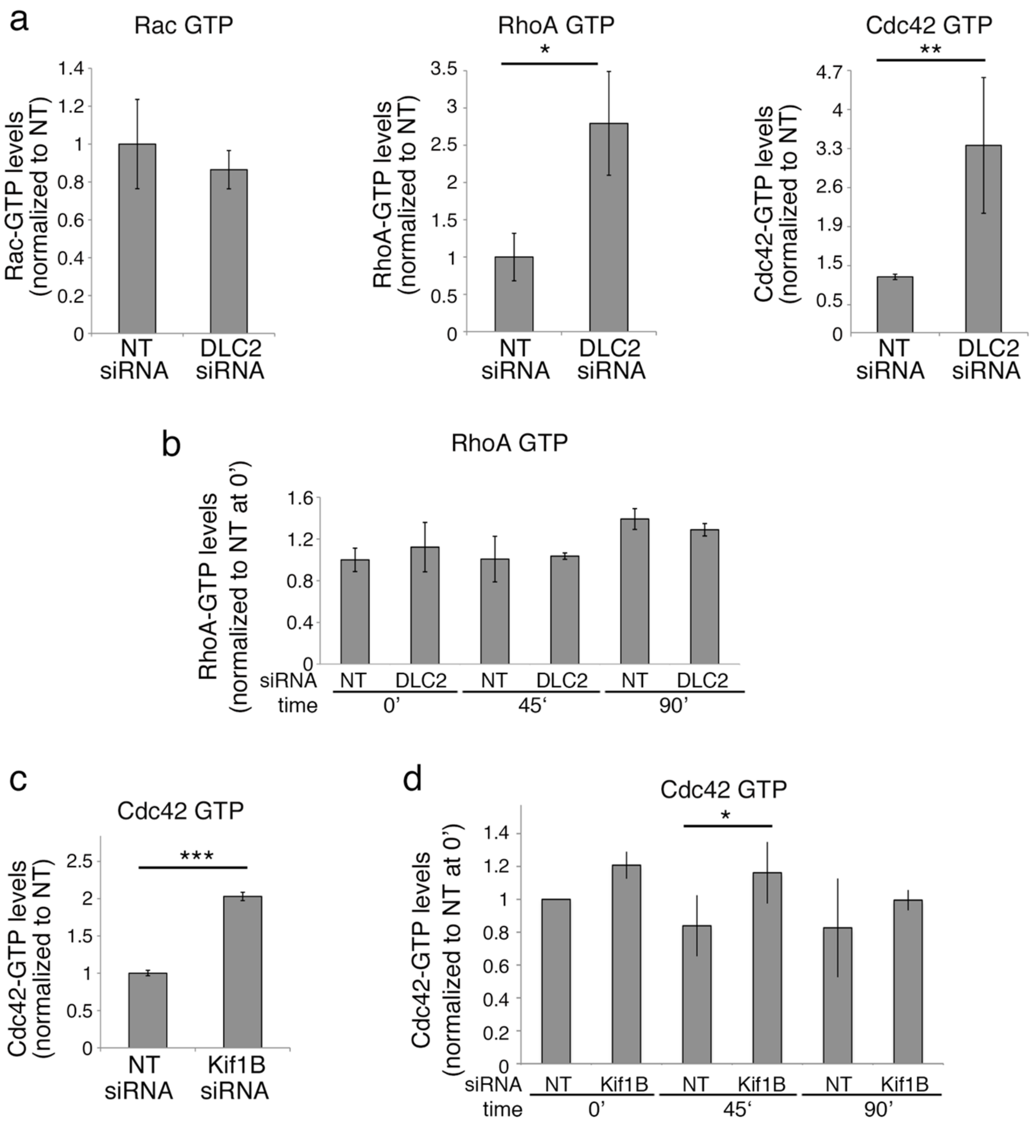

Supplementary Figure 3 Regulation of RhoGTPases.
(**a**) Rac1, RhoA and Cdc42 activity assays in control and DLC2 depleted non-synchronized HCE cells (values were normalized to control cells)**.** (**b**) RhoA activity assays of control (NT, non-targeting) and DLC2 siRNAs transfected HCE cells that had been synchronized with Nocodazole. Time points analyzed after Nocodazole washout were 0', 45' and 90' (shown are means ± 1 SD; n=3 experiments; 45' washout corresponds to metaphase cells; values were normalized to control cells at time 0). (**c, d**) Cdc42 activity analysis of control and KIf1B siRNA transfected cells in interphase (c; values were normalized to control cells) and Nocodazole synchronized cells after washout (d, values were normalized to control cells at time 0). All graphs show means ± 1 SD; n=3 experiments. (* p<0.05, ** p<0.01, *** p<0.001, t-test)

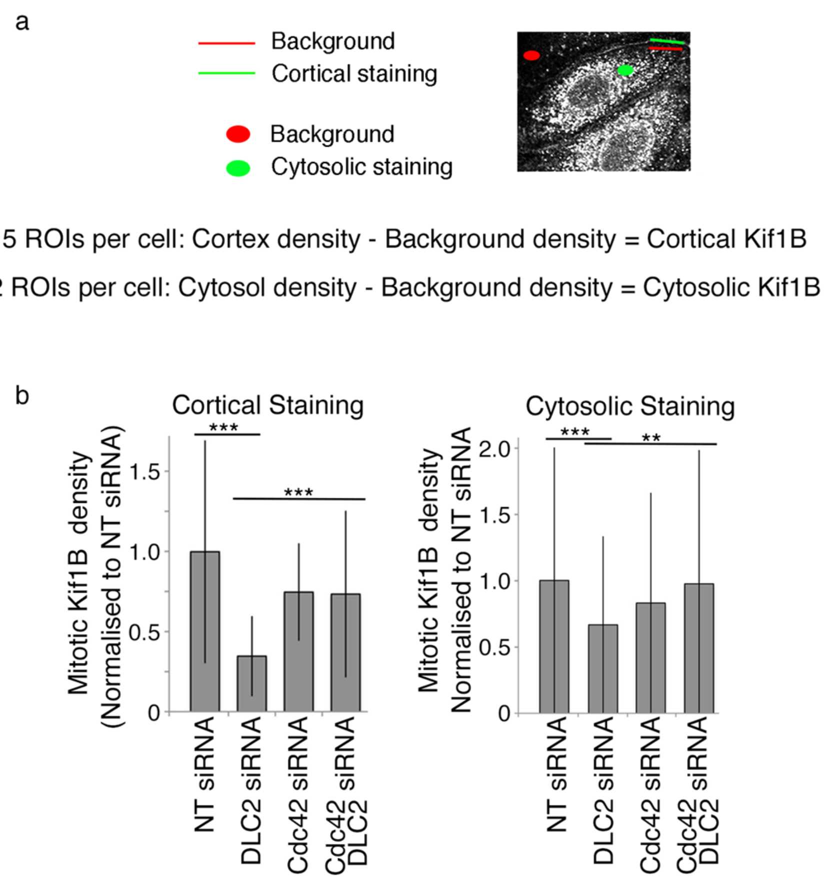

Supplementary Figure 4 Quantification of Kif1B expression.
(**a**) Shown is an image of a cell stained for Kif1B. Such images were acquired at constant microscope settings from samples that had been stained in parallel from the different siRNA transfections. The images were then quantified by measuring mean densities as indicated in the red and green marked regions of interest. Backgrounds (red) were then deducted from the stained junctional and cytosolic areas (green). The values were then normalized to the means of the non-targeting siRNA transfections. (**b**) Quantification of cortical and cytosolic Kif1B staining in cells stained as in figure 4**e** (means ± 1 SD; n=10 cells). (** $p<0.01$, *** $p<0.001$, t-test)

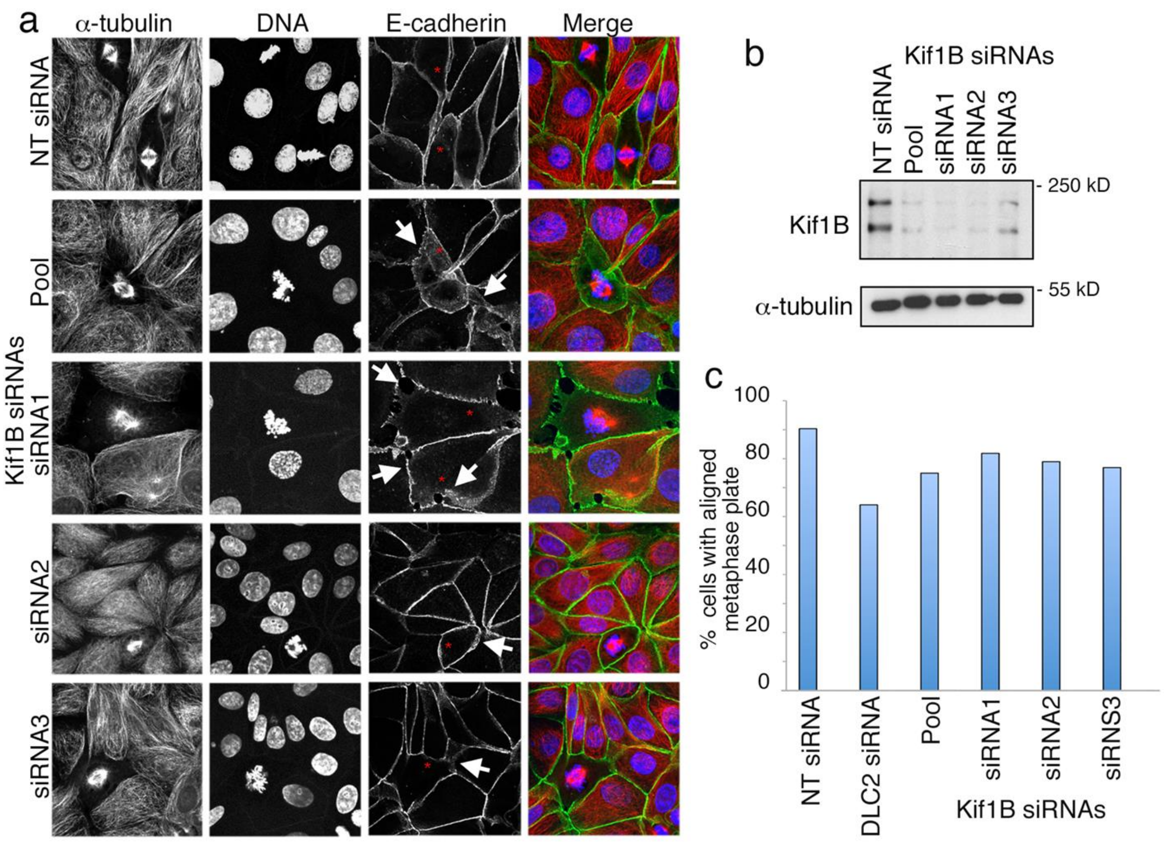

Supplementary Figure 5 Analysis of Kif1B siRNAs.
(**a**) Control and Kif1B siRNA transfected HCE cells were stained for E-cadherin (green), α-tubulin (red) and DNA (blue). Asterisks label mitotic cells and arrows point to dissociated cell-cell junctions. Scale bar, 10 μm (**b**) Immunoblots for Kif1B and α-tubulin of total cell extracts of HCE cells transfected with different Kif1B siRNAs as indicated. (**c**) Quantification of cells with bipolar spindles with aligned and misaligned metaphase plates transfected with control or Kif1B. Shown is a quantification of a deconvolution experiment.

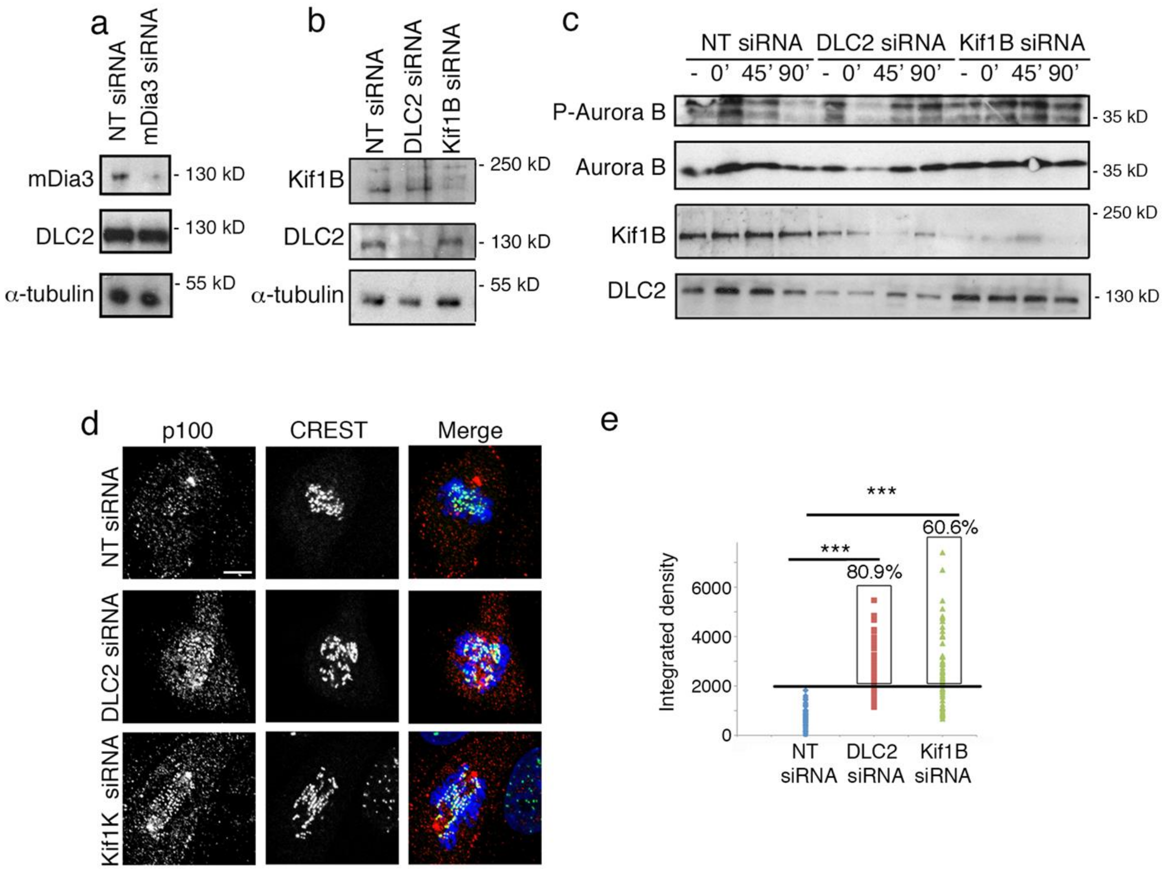

Supplementary Figure 6 DLC2 and Kif1B depletion induced-metaphase delay.
(**a**) Immunoblots of control and mDia3 depleted HCE cells. (**b**) Immunoblots of control, DLC2 and Kif1B depleted HeLa cells; α-tubulin was used as loading control. (**c**) Immunoblots of control, DLC2 and Kif1B depleted HCE cells, treated or untreated with Nocodazole and lysed after 0', 45', and 90' after washout. (**d**) Confocal images of control, DLC2 and Kif1B depleted HCE cells stained for DNA (blue), p100 (phospho-Dsn1, red) and CREST (green). (**e**) Quantification of phospho-Dsn1 staining. Points represent individual cells analysed. The threshold (black line) was identified as the highest integrated intensity in control cells (n=30). (* $p<0.05$, *** $p<0.001$, t-test) Scale bars, 10 μm.

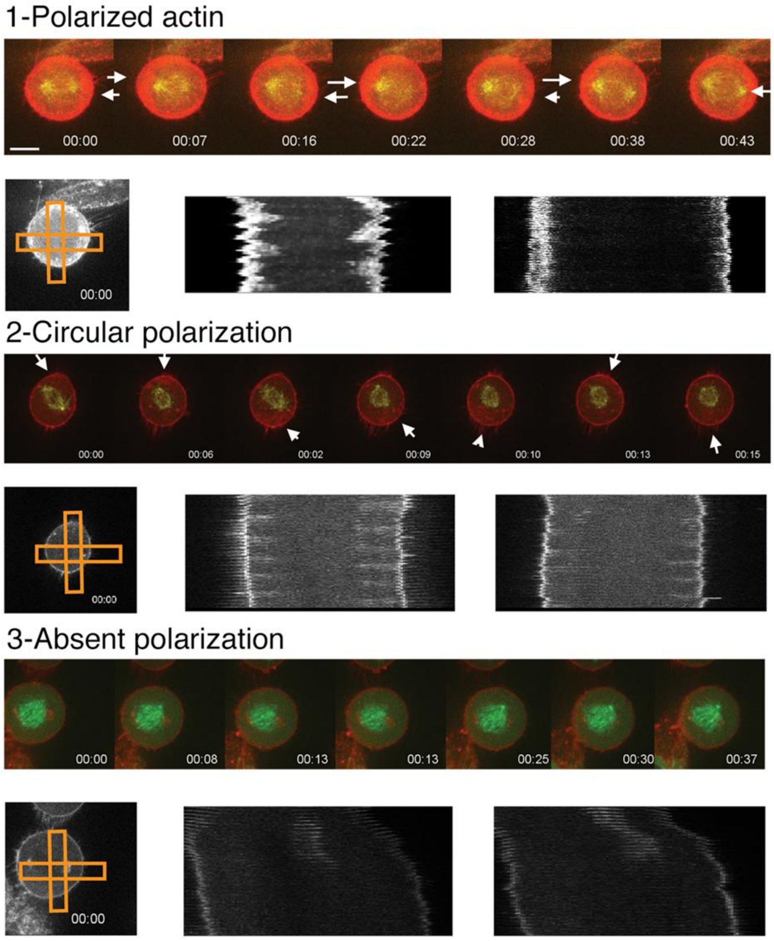

Supplementary Figure 7 DLC2 and Kif1B regulate spindle positioning.
HeLa cells stably transfected with m-Cherry-lifeact and GFP-EB3 were plated onto micropatterned dishes and filmed every 1’ to follow actin behaviour in mitosis. 1- Sequence of pictures for an example of polarized actin behaviour. Two kymographs were drawn, one through the plane of the polarization and one perpendicular, to illustrate actin dynamics. The parallel one shows the alternate left-right shifting; the perpendicular one confirms that there is less F-actin in this area. 2- Sequence of pictures for an example of random polarized actin behaviour. The two kymographs show that actin polarization is similar along both axes. 3- Sequence of pictures for an example of absent polarization. The two kymographs drawn show no clear polarization of F-actin at the cell cortex. Scale bars, 10 μm.

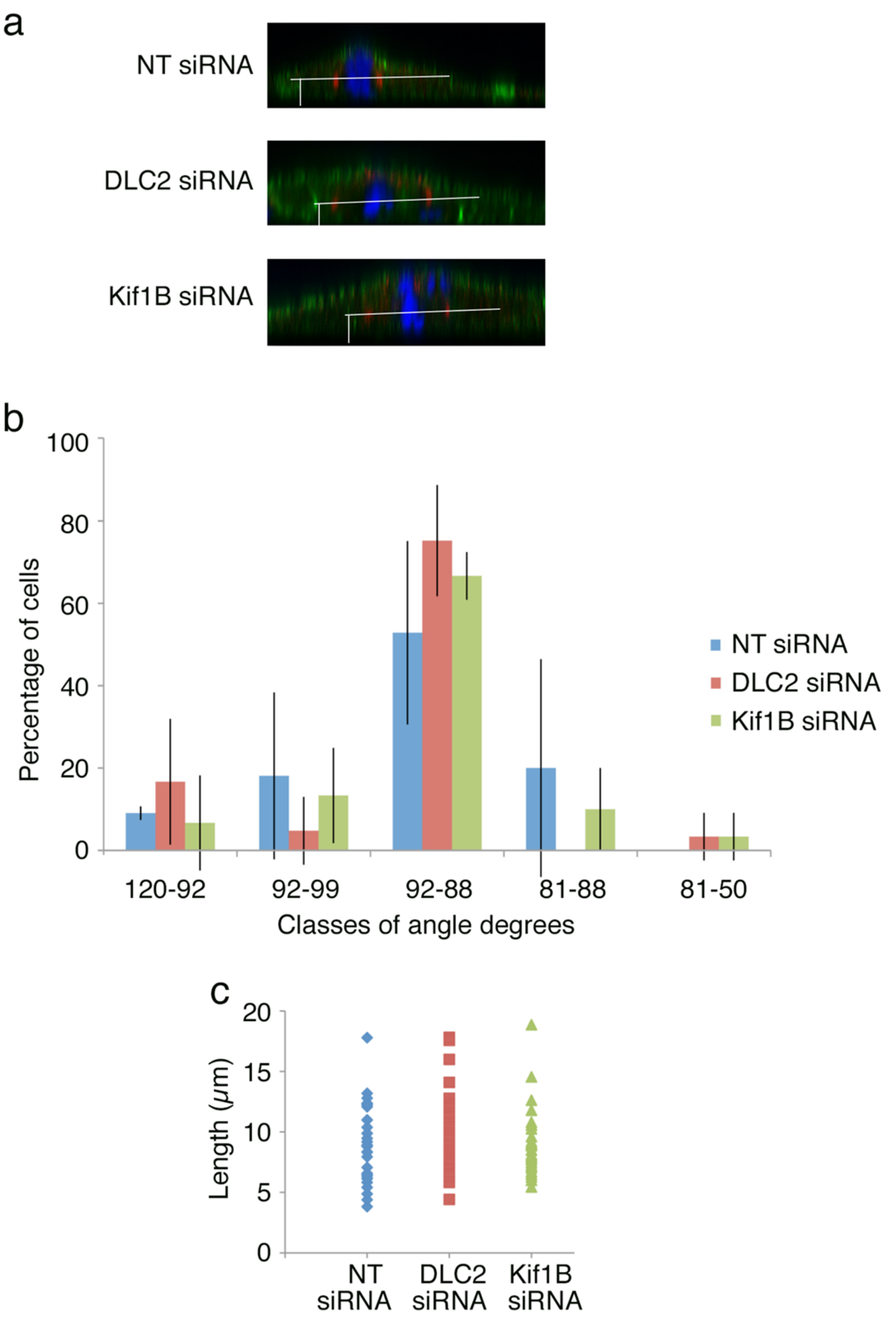

Supplementary Figure 8 DLC2 and Kif1B depletion does not alter the spindle orientation. (**a**,**b**) HCE cells were transfected with siRNAs and then stained for γ-tubulin in red, E−cadherin and DNA in blue. A z-scan was acquired and lines were drawn passing through the two spindle poles and the cell junctions. Spindle angles were calculated in the z-line picture by measuring the angle between a line perpendicular to the substrate and the line passing through the two spindle poles. 5 groups of angles were assigned and are shown in panel b. Shown are means ± 1 SD; n=3 experiments. Over 30 angles per condition were measured. (**b**) The distances from pole to pole are shown as scatter blots. Over 50 cells per condition were measured. Scale bar, 10 μm

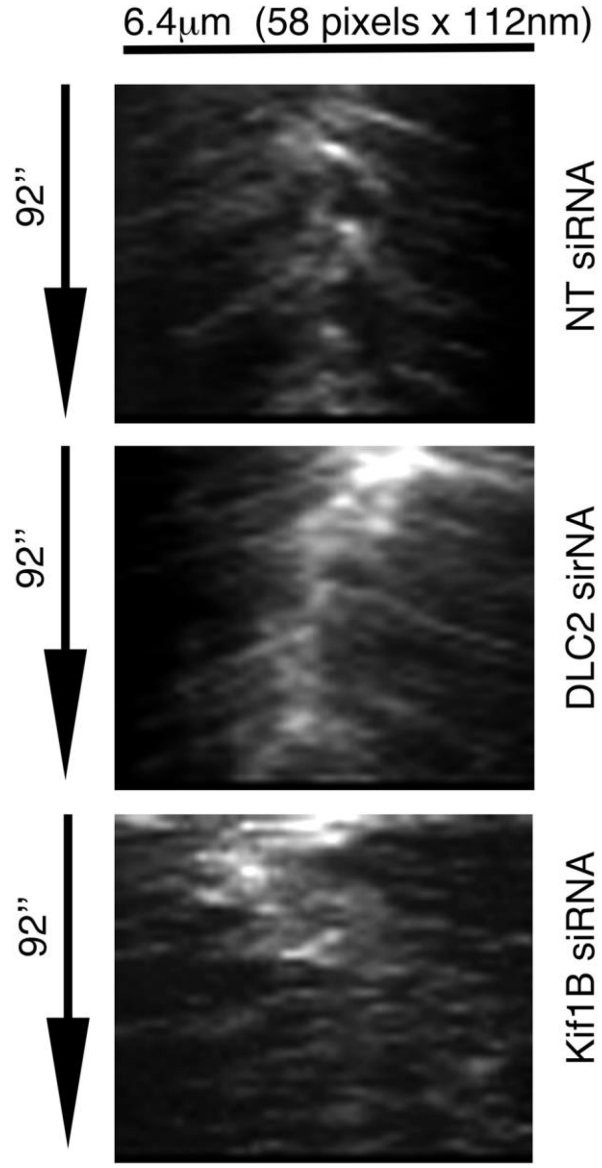

Supplementary Figure 9 Regulation of microtubule length.
HeLa cells stably transfected with GFP-EB3 were filmed every 10''. The behaviour of GFP-EB3 was then tracked. Shown are examples of kymographs used for the quantification.

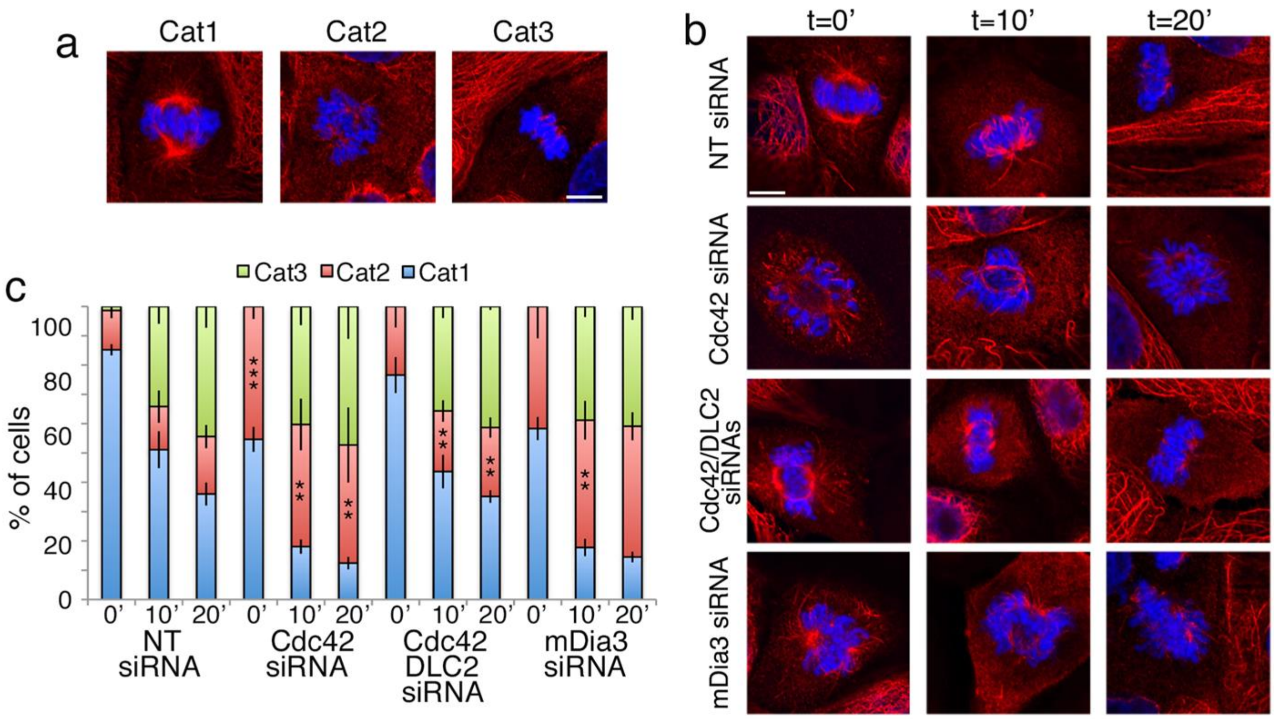

Supplementary Figure 10 DLC2 and Kif1B regulate microtubule dynamics.
(**a**) Categories of cells counted in cold treatment experiments of cells transfected with Cdc42 and mDia3 siRNAs. (**b**) Example panels of the response of control, Cdc42 and mDia3 siRNA transfected cells to cold. (**c**) Chart of percentages of the 3 categories for control, Cdc42 and mDia3 siRNA transfected HCE cells (shown are means ± 1 SD; n=3 experiments). Scale bars, 10 µm. (* p<0.05, ** p<0.01, *** p<0.001, t-test)

Figure 1a

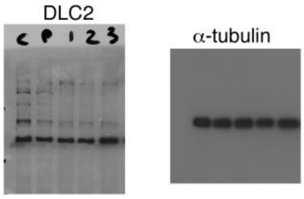

Figure 1g

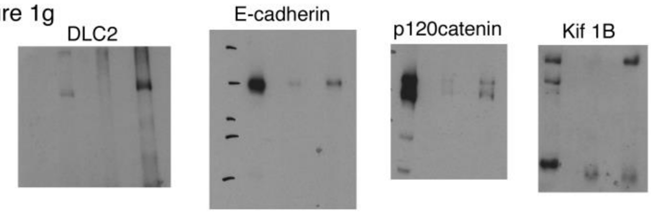

Figure 1h

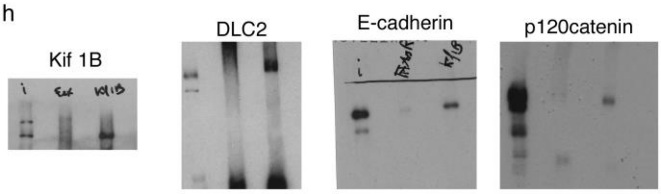

Figure 2e

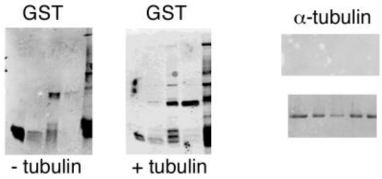

Figure 2f

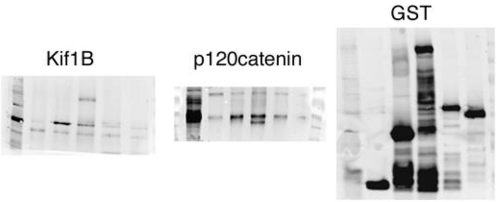

Figure 2g

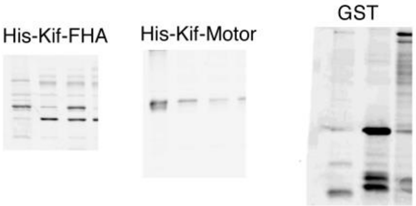

Figure 3e

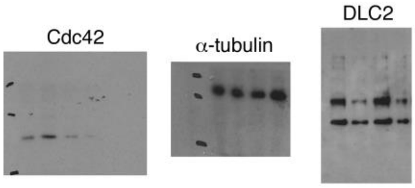

Figure 4c

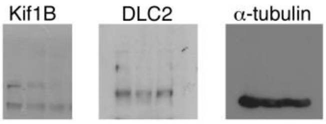

Supplementary Figure 11 Images of immunoblots shown in figurs 1-4
